# Daily Activity Patterns and Overlap Activity of Medium–Large Mammals in Sülüklü Lake Nature Park, Western Black Sea Region, Türkiye

**DOI:** 10.1002/ece3.70654

**Published:** 2024-12-03

**Authors:** Fehmi Yildiz, Ali Uzun

**Affiliations:** ^1^ Department of Biology, Faculty of Science Sakarya University Sakarya Türkiye

**Keywords:** camera‐trapping, large mammals, monitoring, overlapping activity, relative abundance, Türkiye

## Abstract

Türkiye, due to its position as a bridge between Asia and Europe, encompassing three distinct biogeographic regions and its diverse climatic conditions and geographical features, exhibits the characteristics of a small continent in terms of biodiversity, hosting a very high number of mammalian species. However, information on these mammals' activity patterns and co‐occurrence, specifically in Türkiye, is limited. Our study aimed to reveal the daily activity patterns and temporal overlaps of mammalian species detected using camera traps in Sülüklü Lake Nature Park. The white‐breasted hedgehog was strictly nocturnal, while the European badger, gray wolf, European hare, wild boar, and beech marten tended to be nocturnal. The Caucasian squirrel was strictly diurnal, and the roe deer tended to be diurnal. The highest temporal overlap was found between the white‐breasted hedgehog and the beech marten (∆_4_ = 0.84, 95% CI), followed by the red fox and roe deer (∆_1_ = 0.77, 95% CI). The lowest temporal overlap (∆_1_ = 0.081, 95% CI) was found between the white‐breasted hedgehog and the Caucasian squirrel. The second lowest overlap (∆_1_ = 0.136, 95% CI) occurred between the Caucasian squirrel and the European badger. Our findings have provided new and detailed insights into the diversity of mammalian species within the nature park located in Northwestern Anatolia. These data will support and facilitate future research aimed at understanding the mechanisms of species coexistence in this ecosystem. The results obtained will enable a deeper examination of ecosystem dynamics and contribute to developing strategies for biodiversity conservation.

## Graphical abstract

This study investigates terrestrial mammals distributed in and around Sülüklü Lake Naturel Park, Turkey, focusing on their daily activity patterns and temporal overlap. It highlights the biodiversity of the region, revealing significant variations in species' activity, with some showing nocturnal or diurnal behaviors and others exhibiting crepuscular activity. The temporal overlap analysis provides insights into predator–prey dynamics and habitat preferences, influencing species' activity cycles. To promote sustainability and minimize human–wildlife conflicts, the study recommends conservation strategies such as restricting visitor activity during key wildlife periods, establishing buffer zones to reduce disturbances, and encouraging ecotourism practices aligned with wildlife conservation. These findings contribute to a deeper understanding of mammalian behavior in protected areas, emphasizing the importance of sustainable park management for biodiversity preservation.

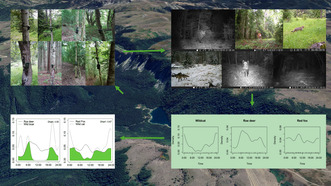

## 1 Introduction

Türkiye, extending along the Anatolian Peninsula and Thrace, encompasses three biogeographic regions: Euro‐Siberia, Mediterranean, and Iran‐Turan, along with their transition zones. Its unique position as a bridge between two continents (Asia and Europe), its proximity to Africa, and its diverse landforms, climatic conditions, and geographical features endow it with the characteristics of a small continent in terms of biodiversity. These environmental characteristics include rapid changes over short distances and hosting numerous distinct ecosystems (Karataş et al. 2021). Türkiye lies at the intersection of three of the 34 global biodiversity hotspots worldwide (Mittermeier et al. 2005). The Mediterranean region alone is home to 321 of the world's mammal species (Temple and Cuttelod 2009), with Türkiye boasting a significant number of 173 mammal species in the region (Karataş et al. 2021).

The investigation of animal activity patterns constitutes an integral facet of behavioral science, providing essential insights into animal behavior (Munro et al. 2006; Yamazaki et al. 2008; Bridges and Noss 2011; Schai‐Braun, Rödel, and Hackländer 2012; Podolski et al. 2013; Lendrum, Crooks, and Wittemyer 2017). Wildlife researchers increasingly deploy camera traps to survey the fauna within a given area, mainly focusing on mammals. This methodology allows for discerning the presence of species, as well as for determining habitat preferences and activity patterns, thereby enabling estimations of both relative and absolute population abundance (Grassman Jr et al. 2006; Giman et al. 2007; McShea et al. 2009). Despite its limitations, particularly when studying arboreal species like squirrels, the employment of camera traps proves invaluable in scrutinizing forest‐dwelling mammalian species characterized by low population densities, nocturnal habits, shyness, and elusive behavior (Gray and Phan 2011).

The circadian rhythms of mammals can be influenced by various factors beyond light cues. These include elements such as the availability of food (Froy 2007; Challet 2010; Ware et al. 2012), social interactions (Favreau et al. 2009), competitive pressures (Vieira and Baumgarten 1995), the presence of predators (Hughes, Ward, and Perrin 1994; Monterroso, Alves, and Ferreras 2013; Caravaggi et al. 2018), and maternal behavior (Duffield and Ebling 1998; Edwards et al. 2021). These variables significantly shape mammals' activity patterns and daily routines, highlighting the intricate interplay between biological, environmental, and social determinants in their behavioral ecology (Mistlberger and Skene 2004). Human activities significantly reduce the spatiotemporal niche of wildlife, limiting the environments and times available for animals to carry out their daily activities and often forcing them to change their natural behaviors to avoid human interaction (Gilbert et al. 2022). Human disturbance alters natural ecosystems by increasing trophic niche overlap among terrestrial carnivores, heightening competition for limited resources (Manlick and Pauli 2020). Environmental determinants also include human‐induced recreational activities. The presence of recreational activities in natural parks can have significant impacts on local wildlife and their habitats (Taylor and Knight 2003; Marzano and Dandy 2012). While these parks are designed to protect and conserve natural ecosystems, the introduction of human activities such as hiking, camping, and wildlife viewing can lead to disturbances in animal behavior and habitat use (Kays et al. 2017). Studies have shown that increased human presence can alter the diel activity patterns of species, often causing them to become more nocturnal to avoid human interaction (Nickel et al. 2020; Procko et al. 2023). Additionally, the infrastructure that supports these activities, such as trails and campsites, can fragment habitats (Ballantyne, Gudes, and Pickering 2014) and further influence species distribution and abundance (Gaines, Singleton, and Ross 2003).

The daily activity patterns of mammals can be categorized into distinct chrono‐ecotypes, including diurnal (day‐active), nocturnal (night‐active), crepuscular (active during dawn and dusk), and cathemeral (equally active during both day and night). It is important to note that these chrono‐ecotypes are not rigid systems. Instead, they can exhibit variability based on specific environmental conditions and needs (Erkert 2008). Diurnal and nocturnal lifestyles are intricate adaptations; what may be beneficial in one system could prove detrimental in another. This underscores mammalian behavior's dynamic and complex nature concerning temporal activities (Kronfeld‐Schor and Dayan 2003).

In this study, our goal was to gain a comprehensive understanding of the medium–large mammal population within Sülüklü Lake Natural Park. Beyond cataloging species, we aimed to explore the daily activity patterns of these mammals and identify potential overlaps in their active periods. This aspect of mammal behavior has not been extensively studied in Türkiye, offering a valuable contribution to the existing research.

## 2 Materials and Methods

### 2.1 Study Area

**FIGURE 1:**
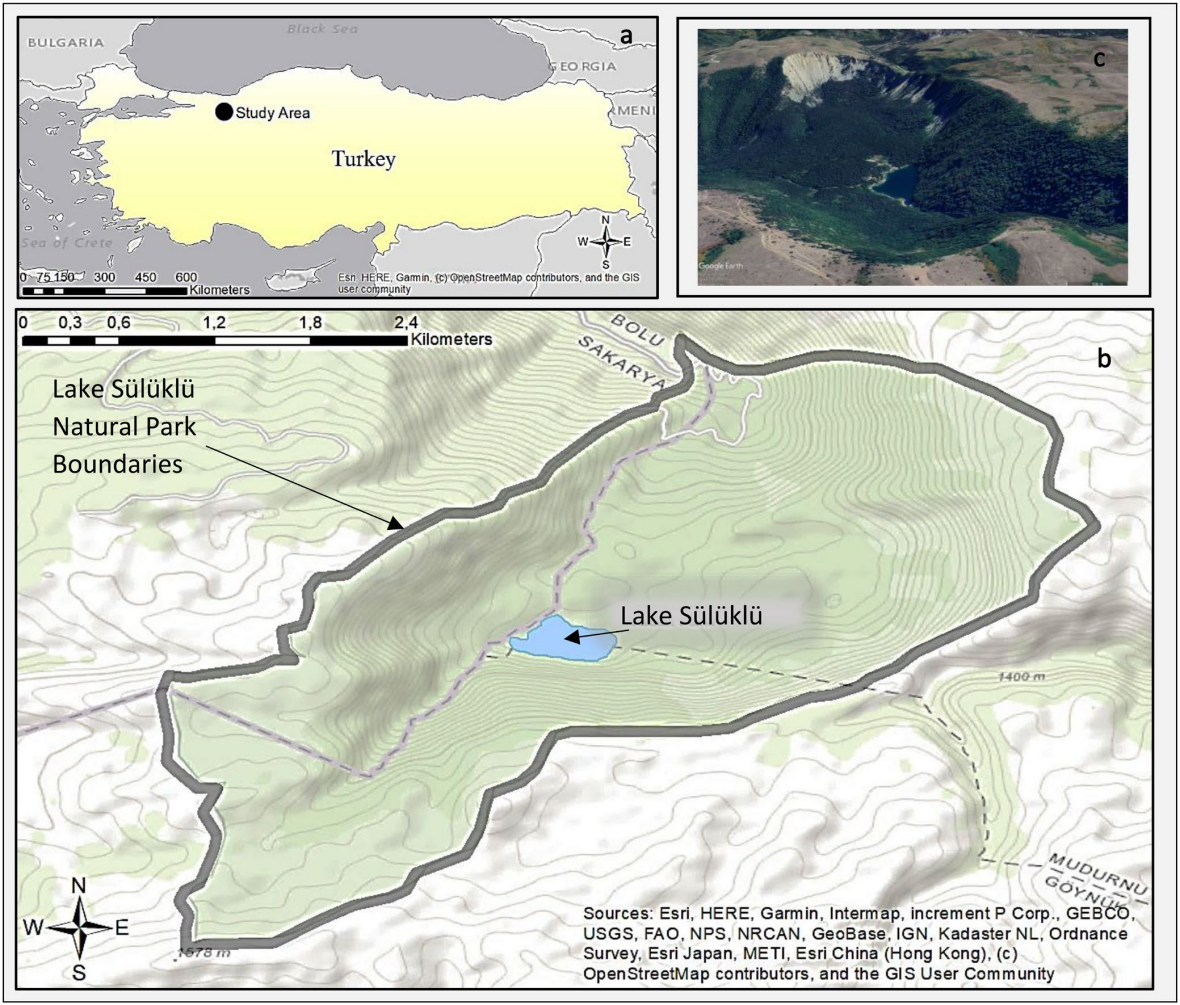
(a) Location of Sülüklü Lake Natural Park in Türkiye (Western Blacksea Region of Türkiye). (b) Sülüklü Lake and Sülüklü Lake Natural Park boundaries. (c) General view of the study area.

Sülüklü Lake Natural Park is located in Bolu province in the Western Black Sea Region of Türkiye (Figure 1). The park encompasses a total area of 810 ha, including Lake Sülüklü, which has a surface area of approximately 60 ha (0.6 km^2^). The lake within the park has a surface elevation of approximately 1070 m, while the surrounding plateaus and hills rise up to 1600 m. In a comprehensive flora study conducted in and around the Park, Kanoğlu, Aksoy, and Kaya (2016) identified 406 taxa belonging to 79 families and 228 genera, 38 of which are endemic. The region is characterized by limy soils due to its calcareous nature, leading to the prevalence of mixed‐leaved forests primarily dominated by Oriental beech (*Fagus orientalis*). One can find Uludag Fir (*Abies bornmuelleriana*) and yellow pine ( *Pinus sylvestris* ) forests on the high mountain slopes. In drier areas of the high mountain slopes comprising trees and shrubs such as black pine ( *Pinus nigra* ) and Türkiye oak ( *Quercus cerris* ), wild pear (*Pyrus elaeagnifolia*), and one‐seed hawthorn (*Crateagus monogyna*), which interact with yellow pine forests. The hills of the high valleys feature dwarf, evergreen shrubs, xeric plants, and scattered common juniper ( *Juniperus communis* ), alongside step‐like flora such as *Astragalus wiedemannianus*, Persian yellow rose ( *Rosa foetida* ), and *Paracaryum incanum*. European box ( *Buxus sempervirens* ) forms extensive communities around the lake, particularly on steep, moist, stony slopes and in forest clearings. In the cooler areas surrounding the lake, cherry laurel ( *Prunus laurocerasus* ), Turkish hazel ( *Corylus colurna* ), and European bladdernut (*Staphylea pinnata*) are frequently encountered and abundant.

Based on the climate data for the region, the coldest months are December and January, with average minimum temperatures dropping to −3°C and average maximum temperatures ranging from 4°C to 6°C. The warmest months are July and August, with average maximum temperatures between 26°C and 27°C and minimum temperatures ranging from 10°C to 14°C (Meteoblue 2024; Appendix A). The lake, formed approximately 300 years ago in 1703 due to a landslide from tectonic movements, still shows traces of these landslides on the surrounding hill's slope (Aytuğ and Kılıç 1986). Given the rarity of this natural event and the unique habitat encompassed by the lake environment, Sülüklü Lake was designated as a protected area in 1988. It retained its status as a wildlife conservation (nature reserve) area for 24 years until 2011, after which, as of that date, it continues to hold the designation of a nature park (Tarimorman 2019). Set amidst a naturally lush landscape, the Park has gained a reputation for offering a wide range of recreational activities, including adventurous pursuits such as trekking, camping, mountain biking, hiking, and mountain running, as well as tranquil pastimes such as picnicking and is open from 08:00 to 21:00, except for campers (Şen 2020).

### 2.2 Camera Trapping

This study was conducted at nine camera trap stations between April and June 2019. The selection of these stations was random, in contrast to a grid‐based approach, owing to the dense forest cover, substantial human activity, and limited access paths within the forest. Trail cameras (Bushnell Trophy Cam E3) were affixed to robust tree trunks using secure belts to minimize movement and positioned 50–100 cm above ground level. Although this height preference is not ideal for arboreal species and species like hedgehogs, a significant number of images of these species were obtained in our study. Camera‐to‐camera distances ranged from 90 to 1200 m. Increasing the number of camera units and placing two cameras instead of one, especially at intervals of 100 m or 150 m, provides a higher detection probability. Moreover, this method is particularly effective for species that are difficult to detect (Evans, Mosby, and Mortelliti 2019). The cameras were configured to record twenty‐second videos at 3‐min intervals (to prevent the same individual from being observed repeatedly and to maintain battery life) between shots, capturing images around the clock. While pictures are more commonly used in camera trap studies and are easier to process, videos offer more detailed insights. Videos have the potential to capture a greater amount of data compared to still images, and their use is expected to grow as technology continues to advance (Swinnen et al. 2014). At each station, efforts were made to clear vegetation blocking the camera's field of view for optimal recording. Checks were made every 2 weeks (to avoid interfering with activity) to replace batteries and maintain records.

### 2.3 Data Analyses

Relative Abundance Index (RAI) is a metric used in ecology to quantify the abundance of a species relative to the total abundance of all species in a particular area or community. It provides a way to compare the density or frequency of different species within a community. RAI was calculated for each species to facilitate comparisons of species densities within the area. The RAI value was determined by dividing the number of species detections by the total number of camera trap days and multiplying the result by 100 (Carbone et al. 2001; O'Brien, Kinnaird, and Wibisono 2003). The independent events in camera trap photographs were defined based on the condition that the interval between two consecutive images of an individual exceeded 10 min (Bogdan, Jůnek, and Jůnková Vymyslická 2016). The recorded data were organized into discrete intervals of 2 hours. Observations were further categorized into diurnal (8:00–18:00 h), nocturnal (20:00–6:00 h), and crepuscular (6:00–8:00 h and 18:00–20:00 h) periods (Maffei et al. 2005; Monroy‐Vilchis et al. 2011). Species exhibiting irregular and sporadic activity patterns were classified as cathemeral (Tattersall 1987; Gómez et al. 2005). In this study, the coefficient of overlapping activity among medium‐sized wild mammals ($\hat{∆}$Þ) was computed utilizing the “overlap” package (Meredith and Ridout 2016). For cases where the sample size was small (*n* < 50), the estimation was conducted using dhat1 (Δ_1_); alternatively, for sample sizes exceeding 75 (*n* > 75), dhat4 (Δ_4_) was employed. This coefficient operates on a scale from 0 to 1, where 0 signifies complete segregation, while 1 indicates total overlap in daily activity patterns. A rigorous assessment was undertaken to validate the accuracy of the overlap coefficient. This involved generating 95% confidence intervals through 2000 simulations, employing nonparametric bootstrap estimators within the R studio environment, as detailed by (Ridout and Linkie 2009). This robust methodology was used to ensure the reliability and accuracy of our findings.

## 3 Results

### 3.1 Species Richness

Over 675 camera trap days, we obtained a total of 967 records. These included 447 instances of targeted mammal species, 68 domestic cats and dogs, 40 humans, 129 birds, 6 unidentified rodents, 50 reimages, and 227 blank images. Throughout the study, we identified 13 mammal species across nine families (Ursidae, Canidae, Mustelidae, Felidae, Cervidae, Suidae, Erinaceidae, Sciuridae, Leporidae), belonging to 5 orders (Carnivora, Artiodactyla, Insectivora, Rodentia, Lagomorpha). The least frequently detected species were the Eurasian lynx (*n* = 1), brown bear (*n* = 5), European badger (*n* = 9), and European hare (*n* = 17). Conversely, the most abundant species were the white‐breasted hedgehog (*n* = 82), red fox (*n* = 80), and beech marten (*n* = 78). Interestingly, the wild boar (*n* = 19), a species often rapidly and intensely detected in camera trap studies, particularly in Türkiye, showed lower prevalence in our research (Table 1). Additionally, outside of the targeted mammalian species, camera traps were primarily triggered by birds (13.34%) and stray dogs (5.08%).

**TABLE 1:** The frequency of mammal species and other triggering factors were detected using camera traps in the Sülüklü Lake Nature Park.

Species	Common name	Number of images	Records (%)	Number of observed sites
*Lynx lynx*	Eurasian Lynx	1	0.10	1
*Ursus arctos*	Brown Bear	5	0.52	3
*Meles meles*	European Badger	9	0.93	2
*Canis lupus*	Gray Wolf	10	1.03	1
*Lepus europaeus*	European Hare	17	1.76	1
*Sus sucrofa*	Wild boar	19	1.96	5
*Canis aureus*	Golden Jackal	29	2.99	6
*Felis silvestris*	European Wildcat	32	3.31	7
*Capreolus capreolus*	Roe Deer	37	3.82	7
*Sciurus anomalus*	Caucasian Squirrel	48	4.96	6
*Martes foina*	Beech Marten	78	8.06	8
*Vulpes vulpes*	Red Fox	80	8.27	8
*Erinaceus concolor*	Southern White‐breasted Hedgehog	82	8.48	6
Domestic dogs		49	5.08	7
Domestic cats		19	1.96	4
Humans		40	4.13	8
Birds		129	13.34	9
Unidentified rodents		6	0.62	3
Blank images (depending on wind, rain, plantshaking, insects, spiders, etc.)		227	23.47	9
Repeating images		50	5.17	9
Total		967	100%	9

### 3.2 Relative Abundance of Species

Hedgehog (12.14) and red fox (11.85) exhibit notably high RAI values, implying that they are likely the most abundant species in the ecosystem. Additionally, beech marten (11.55), caucasian squirrel (7.11), and roe deer (5.48) also demonstrate relatively high RAI values. The Eurasian lynx exhibited the lowest relative abundance index among recorded species, registering at 0.14. The second lowest relative abundance index was demonstrated by brown bear with 0.74. RAI values of other species detected are given in Table 2.

**TABLE 2:** Activity patterns and abundance metrics of mammal species captured in Sülüklü Lake Nature Park.

Species	F (%)	RAI	N	Photo captures (%)			Daily activity category
				Nocturnal	Diurnal	Crepuscular	
*Erinaceus concolor*	18.34	12.14	82	100	0	0	Strictly nocturnal
*Vulpes vulpes*	17.90	11.85	80	43.75	42.50	13.75	Cathemeral
*Martes foina*	17.45	11.55	78	89.74	6.41	3.84	Nocturnal
*Sciurus anomalus*	10.74	7.11	48	0	95.83	4.17	Strictly diurnal
*Capreolus capreolus*	8.28	5.481	37	21.62	43.24	35.13	Diurnal^a^/Cathemeral^b^
*Felis silvestris*	7.16	4.74	32	65.62	25	12.5	Nocturnal^a^/Cathemeral^b^
*Canis aureus*	6.49	4.296	29	68.96	13.79	17.24	Nocturnal^a^/Cathemeral^b^
*Sus sucrofa*	4.25	2.814	19	89.47	0	10.53	Nocturnal
*Lepus europaeus*	3.80	2.518	17	76.47	0	23.53	Nocturnal
*Canis lupus*	2.23	1.481	10	60	30	10	Nocturnal
*Meles meles*	2.01	1.33	9	77.77	0	22.22	Nocturnal
*Ursus arctos*	1.12	0.74	5	100	0	0	Deficient data
*Lynx lynx*	0.22	0.148	1	100	0	0	Deficient data
	100%		447				

Abbreviations: F, Frequency (Numb. of detections of the species × 100/total numb. of detections of all species); N, Number of views; RAI, Relative abundance index.

^a^ Priority activity preference.

^b^ Secondary activity preference.

### 3.3 Activity Patterns

**FIGURE 2:**
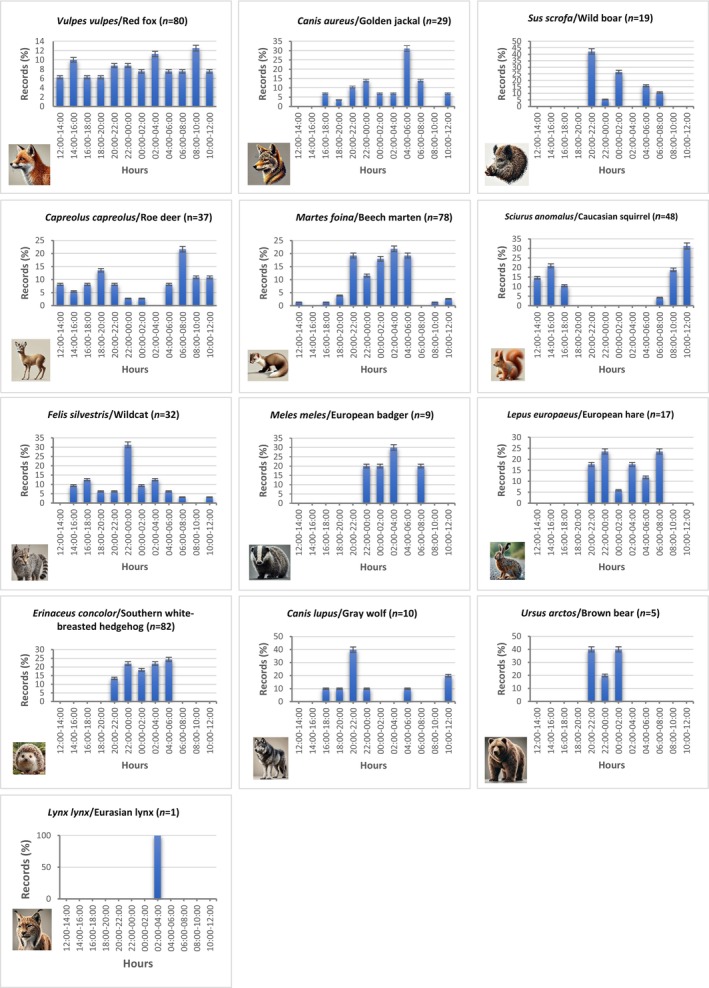
The daily activity frequencies of terrestrial mammal species detected by camera trap method in Sülüklü Lake Nature Park. The density graphs of the species' daily activity patterns are additionally presented in Appendix D.

The hedgehog exhibited unambiguous nocturnal behavior, with all observations (100%) recorded between 20:00 and 06:00. Other species showing nocturnal activity included the beech marten, European badger, wildcat, European hare, and wild boar. The red fox was classified as cathemeral, showing almost equal activity during both day (42.5%) and night (43.75%). The Caucasian squirrel was the only species we detected that exhibited apparent diurnal activity (95.83%). The roe deer showed diurnal activity (43.24%), although crepuscular activity (35.13%) was also observed to a significant extent. Golden jackal (68.96%) and gray wolf (60%) are predominantly nocturnal in their activity patterns; however, they have also been observed to exhibit activity during the day (cathemeral). Despite the records being at night, the daily activity status for brown bear (*n* = 5) and Eurasian lynx (*n* = 1) could not be determined due to the limited number of records available. Detailed distributions of the activity periods of the species we detected in our study are given in Table 2 and Figure 2.

### 3.4 Overlapping Activity

The highest activity overlap coefficient (∆_4_ = 0.844) was observed between hedgehog and beech marten. Following closely, red fox and roe deer exhibited significant activity overlap, with a ∆_1_ value of 0.775. The hedgehog and Caucasian squirrel demonstrated the lowest level of activity overlap (∆_1_ = 0.081) by being rarely observed during the same period. The second lowest level of activity overlap, as indicated the coefficient of ∆_1_ = 0.136, was observed in the interactions between caucasian squirrel and European badger. Among the carnivores we detected, the species with the highest overlap activity were European badger and beech marten, with a value of ∆_1_ = 0.717. The second highest carnivore species activity overlap (∆_1_ = 0.712) was between beech marten and golden jackal.

**FIGURE 3:**
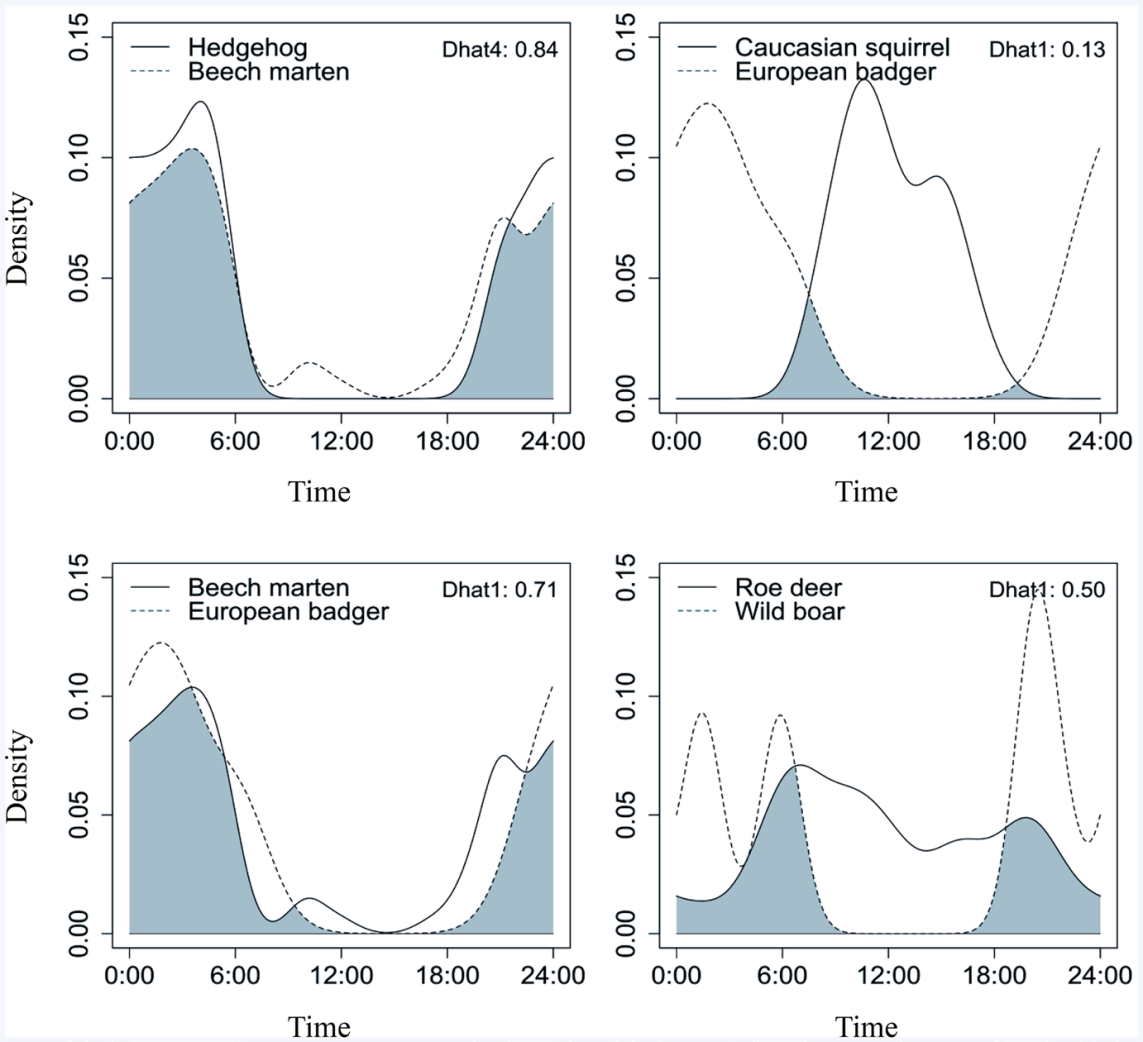
Temporal overlap (painted area) between 11 mammalian species in Sülüklü Lake Nature Park. Each pair overlap coefficient (Δ_1_ or Δ_4_) is given in the upper right corner of the graph (Appendix B).

The lowest overlap activity between carnivore species was observed between European badger and Gray wolf with a coefficient of 0.362. Among the species we detected, only two belong to the order Artiodactyla (Roe deer and wild boar), and the overlap activity value between them is ∆_1_ = 0.501. The activity overlap coefficients between the species Eurasian lynx and brown bear with other species have not been calculated due to the low number of observations for these species. The coefficients for interspecies activity overlap and 95% confidence intervals are presented in Table 3 and Figure 3.

**TABLE 3:** The daily activity overlap coefficients (Dhat1/∆_1_) and confidence intervals for the eleven mammal species in Lake Sülüklü Nature Park. Observation counts exceeded 75 for red fox‐hedgehog, red fox‐beech marten, and hedgehog‐beech marten interactions; hence, the Dhat4/∆_4_ coefficient was used for activity overlap.

Species											
Golden jackal	G.J.										
Gray wolf	0.495 (0.27–0.74)	G.W.									
Roe deer	0.628 (0.36–0.75)	0.558 (0.39–0.78)	R.D.								
Beech marten	0.712 (0.57–0.87)	0.484 (0.28–0.71)	0.487 (0.32–0.60)	B.M.							
European badger	0.635 (0.40–0.87)	0.362 (0.08–0.54)	0.414 (0.18–0.60)	0.717 (0.58–0.97)	E.B.						
Caucasian squirrels	0.286 (0.06–0.34)	0.627 (0.35–0.81)	0.577 (0.37–0.68)	0.158 (0.005–0.15)	0.136 (0.03–0.15)	C.S.					
Southern white‐breasted hedgehog	0.653 (0.50–0.81)	0.404 (0.16–0.58)	0.393 (0.19–0.46)	0.844 (0.79–0.96)	0.754 (0.64–0.91)	0.081 (0.004–0.1)	S.W.B.H				
Red fox	0.694 (0.53–0.84)	0.582 (0.38–0.87)	0.774 (0.68–0.92)	0.604 (0.44–0.69)	0.522 (0.31–0.71)	0.520 (0.38–0.61)	0.515 (0.35–0.56)	R.F.			
European hare	0.696 (0.57–0.92)	0.454 (0.18–0.63)	0.468 (0.29–0.64)	0.736 (0.58–0.93)	0.673 (0.49–0.87)	0.142 (0.04–0.19)	0.747 (0.61–0.90)	0.557 (0.35–0.65)	E.H.		
Wild boar	0.669 (0.42–0.81)	0.555 (0.37–0.78)	0.501 (0.28–0.62)	0.724 (0.49–0.87)	0.603 (0.35–0.82)	0.150 (0.07–0.22)	0.682 (0.43–0.83)	0.579 (0.31–0.62)	0.688 (0.38–0.87)	W.B.	
European wildcat	0.614 (0.41–0.81)	0.625 (0.30–0.79)	0.557 (0.39–0.71)	0.695 (0.53–0.83)	0.568 (0.37–0.76)	0.296 (0.12–0.40)	0.645 (0.50–0.79)	0.670 (0.54–0.83)	0.597 (0.42–0.76)	0.646 (0.32–0.80)	E.W.

## 4 Discussion

### 4.1 Relative Abundance of Species

In our study, the hedgehog was found to have the highest RAI value at 12.14. This finding differs from other studies conducted in various regions of Türkiye, where different species were dominant in terms of abundance. For example, in studies by Çoğal and Sözen (2020), Can (2008), and İlemin and Gürkan (2010), the wild boar had the highest RAI values (8.97, 12.66, and 8.66, respectively). Similarly, Mengüllüoğlu (2010) and Akbaba and Ayaş (2012) reported the European hare as having the highest RAI values (8.59 and 4.78). Soyumert's (2010) study identified roe deer as the most abundant species (28.51), followed closely by wild boar (28.44). In contrast, in our research, wild boar ranked in the middle range with an RAI of 2.81, demonstrating a notable difference from the studies mentioned earlier. These divergent findings highlight the distinct ecological dynamics within our study area, pointing to potential differences in habitat structure, food resource availability, or anthropogenic influences. By reporting a higher abundance of hedgehogs, our research offers fresh insights into species distribution patterns in Türkiye, distinguishing it from prior studies and contributing new perspectives on mammalian abundance in the region.

Habitat variation, climatic conditions, human influences, research methodologies, and seasonal shifts in species habitat use contribute to the variability of RAI values across geographic regions (Foster and Harmsen 2012; Sollmann et al. 2013). Various habitat types and structures can influence species abundance, while climatic conditions and human activities can exacerbate this influence (Wearn and Glover‐Kapfer 2017). Additionally, the use of diverse research methods and protocols across studies can lead to variations in results (O'Brien 2011). Furthermore, fauna abundance can vary seasonally and annually, contributing to differing study outcomes (Foster and Harmsen 2012).

In addition to these factors, another element that may affect the relative abundance of species in our study area is the presence of stray dogs, which rank fourth in our detection records. Stray dogs can compete with native species, alter hunting behaviors, and potentially spread diseases, resulting in negative impacts on local biodiversity and posing significant threats to ecosystem balance (Zanin et al. 2019). The increasing number of domestic dogs interacts significantly with native species (natural species) found in natural areas, threatening the abundance of these species (Paschoal et al. 2012). These factors contribute to the diversity of RAI values in studies conducted across different regions.

### 4.2 Activity Patterns

*Erinaceus concolor* , The European hedgehog ( *Erinaceus europaeus* ), is a nocturnal insectivore known for its solitary nature and hibernation habits, thriving in a variety of habitats (Dowding et al. 2010). The majority of the species detected in our study showed nocturnal activity. The collected data set made the most frequently recorded species. The daily activity pattern observed for hedgehogs in the study area is strictly nocturnal. The predominant activity periods were observed during the nighttime, specifically between 22:00–00:00 and 02:00–06:00.

*Vulpes vulpes* , during our investigation, the red fox manifested two distinct peaks of activity, observed between 02:00 to 04:00 and 08:00 to 10:00. Despite these specific peaks, the classification of the red fox as cathemeral was warranted, as it consistently exhibited activity approximately throughout all periods of the day. In previous studies (Blanco 1986; Phillips and Catling 1991; Cavallini and Lovari 1994; Díaz‐Ruiz et al. 2016; Noor et al. 2017), red foxes were found to show predominantly nocturnal and crepuscular activity and were classified as facultative (adaptive) nocturnal by Monterroso, Alves, and Ferreras (2014). The findings of our study, however, demonstrate the potential for red foxes to remain active throughout the day, highlighting a notable difference from the predominantly nocturnal classifications in existing literature. Changes in the daily activity patterns of red foxes can be influenced by a complex set of ecological factors, including food resources (Contesse et al. 2004; Panek and Budny 2017; Reshamwala et al. 2018), predator presence (Levi and Wilmers 2012; Newsome et al. 2014; Gil‐Fernandez et al. 2020; Rees et al. 2024), prey behavior (Monterroso, Alves, and Ferreras 2013; Díaz‐Ruiz et al. 2016; Gil‐Fernandez et al. 2020; Jasiulionis and Balčiauskas 2021; Nardotto 2022), human activity (Baker et al. 2007; Díaz‐Ruiz et al. 2016; Alexandre et al. 2020; Kämmerle, Rondeaux, and Storch 2020; Castro et al. 2022), seasonal changes (Molsher, Gifford, and McIlroy 2000; Kämmerle, Rondeaux, and Storch 2020; Fleming et al. 2021), and habitat structure (Gosselink et al. 2003; Díaz‐Ruiz et al. 2016; Castro et al. 2022). In our study area, the tendency of red foxes to exhibit increased daytime activity is thought to be driven primarily by two factors: avoiding larger nocturnal predators such as the brown bear, European lynx, and golden jackal, and taking advantage of food waste left by visitors to the park. Together, these factors illustrate the red fox's behavioral flexibility and adaptability across diverse ecological settings.

*Lepus europaeus* , observations from our study revealed that European hares exhibited nocturnal and crepuscular activities within specified intervals, showcasing heightened activity post‐sunset (22:00 to 24:00) and pre‐sunrise (06:00 to 08:00). Significantly no diurnal activity was documented during daylight hours. They also showed activity for almost half the day, with an activity period of 12 h (24:00–08:00). As a result of the analysis of 10 studies on rabbit activity patterns, it was reported that the activity of European hares was at the highest level between 20:00 and 05:00 and the minimum level between 10:00 and 15:00 (Mori et al. 2022). These results coincide with the results of our study.

*Felis silvestris* , According to previous European wildcat activity studies, Migli et al. (2021) reported that male wildcats exhibit a typical nocturnal/crepuscular period and show activity with distinct activity peaks at dawn and dusk. Can, Kandemi̇r, and Togan (2011) said that diurnal (06:00–18:00, 50.4%) and nocturnal (18:00–06:00, 49.6%) activities are almost equal in the daily activities of wildcats. In our study, the activity of wildcats peaked between 22:00–00:00 (31.25%), and they showed activity almost at all hours of the day. We classified wildcats as primarily nocturnal and secondarily cathemeral.

*Martes foina* , The beech marten exhibits primary activity during the nocturnal period (Genovesi 1993; Herrmann 1994; Monterroso, Alves, and Ferreras 2014; Bischof et al. 2014; Torretta et al. 2017). Beech martens displayed a predominantly nocturnal (85%) activity pattern, with two activity peaks between 21:00–23:00 and 01:00–03:00 (Roy et al. 2019). Our study revealed that beech martens exhibited a peak in activity (~22%) between 02:00–04:00, demonstrating a substantial predominance of nocturnal activity at a significant proportion of 89.74%. This concentration of nocturnal behavior, particularly during the mentioned time frame, highlights a noteworthy aspect of their activity pattern.

*Meles meles* , In our study, European badgers demonstrated heightened levels of nocturnal (77.77%) and crepuscular (22.22%) activity, indicating significant movement during the night and dawn/dusk periods, with no diurnal activity recorded. These findings align with previous studies, where European badgers exhibited similar nocturnal activity patterns. For instance, Kowalczyk et al. (2003) found that more than 80% of badger activity occurred between 20:00 and 03:00 during spring to autumn. Similarly, Do Linh San (2004) reported a distinct peak in activity, reaching 93.9% between 21:00 and 21:59, with a low of 8.7% recorded between 13:00 and 13:59. Seasonal differences were also noted by Cabré (2020), who observed higher activity before midnight in winter and spring, with more prolonged activity in summer and autumn, peaking around midnight. Our results support these findings, reinforcing badger activity's predominantly nocturnal and crepuscular nature, with clear seasonal trends.

*Capreolus capreolus* , In our study, the activity patterns of roe deer were consistent with previous findings, showing a pronounced bimodal trend with peaks at dawn and dusk. The highest activity was observed between 06:00–08:00 (~22%) and 18:00–20:00 (~14%), while the lowest activity levels were recorded between 22:00–04:00 (~5%). This diurnal/crepuscular activity accounted for approximately 79% of total activity. These findings align with studies such as Stache et al. (2013), who reported that female roe deer are most active at sunrise and sunset, with daytime activity diminishing in early July. Additionally, Krop‐Benesch et al. (2013) noted peak activity at dawn, along with additional peaks in the afternoon and dusk, and Pagon et al. (2013) also described the consistent bimodal activity pattern throughout all seasons, with prominent peaks at dawn and dusk.

*Sciurus anomalus* , In our study, Caucasian squirrels demonstrated clear diurnal behavior, with no recorded nocturnal activity (0%) between 18:00 and 06:00. The activity peaked at 31.25% between 10:00 and 12:00, following a steady increase from 06:00 in the morning, which gradually decreased toward the evening, ending by 18:00. This diurnal pattern aligns with previous research. Ognev (1963) and Harrison and Bates (1991) highlighted the diurnal tendencies of these squirrels. Abi‐Said et al. (2014) observed that red squirrel activity initiated at 06:00, peaking between 09:00–14:00. Gavish (1993) reported similar findings in Israel, where activity persisted until 18:00, and Amr et al. (2006) and Hecht‐Markou (1994) noted continued activity until 19:00 in Jordan and Greece, respectively. Our findings further support the consensus that red squirrels exhibit strong diurnal behavior, with 95.83% of their activity occurring during daylight hours.

*Sus scrofa* , In our study, wild boars displayed predominantly nocturnal activity, with 89.47% of our detections occurring between 20:00 and 06:00, and 10.53% classified as crepuscular. No diurnal activity was observed. These findings align with previous research. Stolle et al. (2015) recorded 59.1% of wild boar detections during the nocturnal period, with peak sightings at 24:00 and approximately 50% of observations occurring between 20:00 and 24:00. Similarly, Russo, Massei, and Genov (1997) reported that wild boars in their native Italian habitats were most active between 17:00 and 07:00. Studies on wild boars in invasive regions have shown varied activity patterns, influenced by factors such as human hunting, which has been linked to both increased nocturnal and diurnal activities (Keuling, Stier, and Roth 2008). The temporal distribution of nocturnal activities, particularly feeding and movement, typically peaks between 00:00–05:00, with a maximum at 02:00–03:00 (Cahill, Llimona, and Gràcia 2003), closely matching the patterns observed in our research.

*Canis lupus* , Gray wolves display diverse activity patterns that demonstrate a level of predictability spanning seasons, locations, and individual behaviors (Packard, Mech, and Boitani 2003). Gray wolves displayed continuous activity during the day; however, their most heightened activity level occurred at dawn and dusk, aligning with the periods when they were most successful in hunting prey (Theuerkauf et al. 2003). Gray wolves exhibited dual peaks in activity, with high points at both dawn and dusk, showcasing increased nocturnal engagement compared to daylight hours (Torretta et al. 2016; Rossa, Lovari, and Ferretti 2021). Throughout our study, gray wolves demonstrated 60% nocturnal activity, with the peak occurring between 20:00 and 22:00.

*Canis aureus* , the golden jackal, is predominantly crepuscular and nocturnal, with reported activity observed throughout the day (Majumder et al. 2011; Katuwal and Dahal 2013; Gupta et al. 2016; Ojha, Sharma, and Rajpurohit 2017). Majumder et al. (2011) findings indicated that the Golden Jackal demonstrated two primary activity peaks in India: one occurring during the early morning hours (04:01–08:00) and the other at night (20:01–00:00). According to a study conducted in Thailand (Charaspet et al. 2019), jackals were observed to be primarily active during the night and twilight hours, with two main activity peaks recorded after sunset and before sunrise. Similarly, Debata (2021) noted in India that golden jackals exhibit predominantly nocturnal and crepuscular behavior, displaying two major activity peaks: one in the late evening after sunset and the other during the early morning until sunrise. The activity pattern exhibited two peaks in the early morning (06:00–10:00) and in the afternoon during the rainy season (16:00–18:00). Resting was more prominent during 10:00–11:00 and 14:00–15:00. In the dry season, the time spent for foraging decreased, corresponding to an increase in resting during the noon hours (10:00–14:00). However, shallow resting patterns were recorded between 06:00–8:00 and 16:00–18:00, as reported by Gashe and Yihune (2020). In congruence with earlier studies, our research indicates nuanced activity patterns in jackals, showing a predominant distribution of 68.96% in nocturnal activity, 17.24% in crepuscular activity, and 13.79% in diurnal activity.

Discrepancies in daily activity patterns observed across different studies may stem from various factors, encompassing geographical distinctions, climatic variations, and environmental conditions, including the impact of human activities (Ferreguetti, Tomás, and Bergallo 2015; Gaynor et al. 2018). Moreover, variations in sunset and sunrise times and differences in the duration of daylight and nighttime can contribute to these differences (Daan and Aschoff 1982; Ensing et al. 2014). Additionally, the availability or scarcity of other animal species in a particular region can further influence and account for the documented variations in daily activity patterns (Kronfeld‐Schor and Dayan 2003; Sollmann et al. 2013). The complex interplay of these ecological, environmental, and temporal factors underscores the need for a comprehensive understanding when interpreting and comparing findings across diverse studies (O'Brien and Kinnaird 2011; Foster and Harmsen 2012).

### 4.3 Overlapping Activity

Our findings perfectly align with those of Tsunoda et al. (2020) in the Stara Planina Mountains of Bulgaria, as the overlap activity value between the golden jackal and wildcat was identical at ∆ = 0.61. For the gray wolf and European badger, our study revealed the lowest overlap value at 0.36 compared to other studies (Ferretti et al. 2023 (0.75–0.86); Mori et al. 2020 (0.78); Ogurtsov, Zheltukhin, and Kotlov 2018 (0.63); Rossa, Lovari, and Ferretti 2021 (0.85)). Similarly, the overlap between gray wolf and wild boar in our research was ∆ = 0.55, also lower than in other studies (Mori et al. 2020 (0.88); Ogurtsov, Zheltukhin, and Kotlov 2018 (0.78); Rossa, Lovari, and Ferretti 2021 (0.81)). Interestingly, our study recorded a higher coefficient of temporal activity overlap between golden jackal/beech marten (∆ = 0.71) and golden jackal/European badger (∆ = 0.63) compared to Tsunoda et al. (2018, 2020). The temporal overlap coefficient between European hare and roe deer in Viviano et al.'s (2021) Italian study was 0.81, nearly double our finding of 0.46. For red fox and European badger, the overlap in our study was at its lowest, with a value of 0.52, lower than previous studies (Mori and Menchetti 2019 (0.90); Mori et al. 2020 (0.77); Tsunoda et al. 2020 (0.74); Zalewska et al. 2021 (0.64)). Among ungulates, the overlap between wild boar and roe deer was 0.50, the lowest reported compared to other studies (Mori et al. 2020 (0.78); Zanni et al. 2021 (0.63)). Finally, Franchini et al. (2022) reported a range of 0.44 to 0.82 in overlap between red foxes and wildcats across various Italian habitats, whereas our research yielded a value of 0.67.

The fundamental reasons for the variation in temporal overlapping among mammalian species across different geographical regions, and even within the same geographic regions, include habitat differences, food resources and competition, climate and seasonal variations, anthropogenic effects, population dynamics, relative abundance, activity patterns, and predator–prey relationships (Monterroso, Alves, and Ferreras 2014; Frey et al. 2017; Cohen, Lajeunesse, and Rohr 2018; Gaynor et al. 2018; Tucker et al. 2018; Santicchia et al. 2018; Tudge et al. 2022). The diversity and competition level of food resources in different geographic regions can alter the interactions between species, including predator–prey relationships (Sih, Ferrari, and Harris 2011; Foster et al. 2013; Moll et al. 2018). These factors, along with relative abundance and activity patterns, influence the species' habitats, feeding habits, and adaptation capacities, thus leading to variations in temporal overlapping (Foster et al. 2013; Gaynor et al. 2018). The dynamics of predator–prey relationships also play a crucial role in shaping the temporal overlap among mammalian species (Ripple and Beschta 2004; Foster et al. 2013; Suraci et al. 2016; Gaynor et al. 2019). These intricate interactions result in different outcomes of temporal overlapping among mammalian species in various geographical regions (Monterroso, Alves, and Ferreras 2014; Gaynor et al. 2018). Therefore, comprehensive research considering multiple factors, including predator–prey relationships, is necessary to understand the interactions and temporal overlapping among mammalian species (Tucker et al. 2018; Gaynor et al. 2018). Comparative information on interspecies temporal activity overlap, including previous studies and the results of this research, is provided in detail in Appendix C.

## 5 Suggestions

Firstly, Park management should regularly monitor wildlife behavior and assess areas to identify potential threats (Sutherland et al. 2018). Park visitors should be informed about the species of wild mammals they may encounter, educated about their natural behaviors, and provided with information. This can help minimize human–mammal conflicts (Newsome, Dowling, and Moore 2005; Ballantyne, Packer, and Hughes 2009). Proper management of food leftovers and garbage can reduce conflicts in areas frequented by wild mammals (Herrero and Higgins 2003; Baruch‐Mordo et al. 2008). Restricting visitor‐related food sources can significantly minimize the likelihood of interactions between wild mammals and humans (Spencer, Beausoleil, and Martorello 2007). Preserving and restoring natural habitats can assist in conserving the living spaces of wild mammals, supporting species spread, and enhancing the effectiveness of conservation measures (Ripple and Beschta 2012; Laurance et al. 2014; Haddad et al. 2015). Particularly around the lake and its surroundings, where visitors spend time, physical barriers like fences can limit contact between wild mammals and humans (Buckley 1991). If there is an excessive increase in wild mammal populations in specific areas, habitat modifications or management interventions may be necessary to maintain balance and prevent potential conflicts (Côté et al. 2004; Ripple and Beschta 2012).

Park managers can develop regular research and monitoring programs to track wild mammal populations and behaviors, which are crucial for developing effective conservation strategies (Gibbs, Snell, and Causton 1999). Furthermore, trekking routes, a popular recreational activity in the area, should be positioned away from areas with dense wild mammal populations and wildlife trails as much as possible (George and Crooks 2006; Marion, Leung, and Nepal 2006; Larson et al. 2016). This can contribute to the conservation of natural habitats and visitor safety (Taylor and Knight 2003; Reed and Merenlender 2008; Hammitt, Cole, and Monz 2015). Warning signs about the presence of wild mammals should be placed along trekking routes to encourage visitors to be cautious and avoid disturbing wildlife. Guided tours can be offered along trekking routes, providing visitors with information on how to behave if they encounter wild mammals (especially if brown bears are spotted in the area). Emergency safety protocols should be established, and visitors should be informed about them. Information on what to do in case of encounters with wild mammals is essential for ensuring visitor safety. Nature observation areas can be established at various points along trekking routes. These areas can offer visitors opportunities to observe and photograph wild mammals without disturbing natural habitats. Adequate facilities should be provided along trekking routes for visitors to properly manage their garbage and waste. This can prevent wild mammals from accessing waste sources and thus reduce human–mammal conflicts.

In recent years, the increasing stray dog problem in Türkiye has posed a significant threat to wildlife as well. Our study shows that stray dogs have been detected at a high rate in the area. Numerous studies have been conducted on the adverse effects of stray dogs on wildlife (Butler, du Toit, and Bingham 2004; Suzán and Ceballos 2005; Lacerda, Tomas, and Marinho‐Filho 2009; Young et al. 2011; Hughes and Macdonald 2013; Lessa et al. 2016; Home, Bhatnagar, and Vanak 2018; Oğurlu et al. 2022). Domestic dogs have been implicated in the extinction of 11 vertebrate species, posing a known or potential threat to at least 188 threatened species globally (Doherty et al. 2017). In this context, park managers should regularly conduct inspections in park areas to identify the presence of stray dogs and intervene immediately. This can ensure the safety of the park and the preservation of wildlife. Clear rules and legal regulations regarding the bringing and releasing pets in parks should be established. Penalties should be imposed for violations of these regulations. Park boundaries should be defined, and pet owners should not be allowed to release their animals within these boundaries. Additionally, signs indicating these rules should be placed at park entrances.

## 6 Conclusion

This study provides valuable insights into the daily activity patterns and temporal overlap of medium and large mammals in Sülüklü Lake Nature Park. Notably, a high degree of activity overlap was observed between the hedgehog and the beech marten, while the Caucasian squirrel and the European badger exhibited minimal overlap. These findings highlight significant differences in temporal niche utilization among species, offering critical insights into coexistence mechanisms within the ecosystem. However, the study is limited by its timeframe and the small sample sizes for certain species, such as the Eurasian lynx and brown bear, which prevented definitive conclusions about their activity patterns. Additionally, the effects of seasonal variations were not fully explored. Future research should focus on long‐term studies across different habitats to better understand the seasonal influences on species' activity. Moreover, a deeper investigation into predator–prey relationships and the impact of human activities on mammalian behavior is essential. Such studies would provide critical information for species conservation and effective ecosystem management strategies in Türkiye.

## Author Contributions

**Fehmi Yildiz:** conceptualization (lead), data curation (lead), investigation (lead), methodology (lead), visualization (lead), writing – original draft (lead). **Ali Uzun:** conceptualization (supporting), investigation (supporting), methodology (supporting).

## Conflicts of Interest

The authors declare no conflicts of interest.

## Supporting information


Data S1.

Data S2.

## Data Availability

The data and codes used in this study have been uploaded as supporting information.

## Appendix C Comparisons of Overlap Activity Analysis Results in this Study and Previous Mammals Activity Studies

**Table:**

Interspecies	Country	Study area	Mainly landscape	Overlap coefficient	References and this study
European badger‐Beech marten	Bulgaria	Stara Planina Mountains	Secondary oak forests	0.83 (0.75–0.90)	Tsunoda et al. (2020)
European badger‐Beech marten	Türkiye	Sülüklü Lake Nature Park	See: Study area	0.71 (0.58–0.97)	This study
European badger‐Wild boar	Russia	Central Forest State Nature Bio. Res.	Poor forest areas	0.54 (0.47–0.62)	Ogurtsov, Zheltukhin, and Kotlov (2018)
European badger‐Wild boar	Türkiye	Sülüklü Lake Nature Park	See: study area section	0.60 (0.35–0.82)	This study
European badger‐Wild cat	Italy	Pollino National Park	Deciduous woodland‐lowlands	0.71 (0.68–0.74)	Mori et al. (2020)
European badger‐Wild cat	Bulgaria	Stara Planina Mountains	Secondary oak forests	0.77 (0.69–0.85)	Tsunoda et al. (2020)
European badger‐Wild cat	Türkiye	Sülüklü Lake Nature Park	See: study area section	0.56 (0.37–0.76)	This study
European hare‐Roe deer	Italy	Southern Tuscany	Deciduous woodlands	0.81 (0.77–0.88)	Viviano et al. (2021)
European hare‐Roe deer	Türkiye	Sülüklü Lake Nature Park	See: study area section	0.46 (0.29–0.64)	This study
Golden jackal‐Beech marten	Bulgaria	District of Stara Zagora	Farmland and forest	0.50 (0.37–0.68)	Tsunoda et al. (2018)
Golden jackal‐Beech marten	Bulgaria	Stara Planina Mountains	Secondary oak forests	0.55 (0.47–0.62)	Tsunoda et al. (2020)
Golden jackal‐Beech marten	Türkiye	Sülüklü Lake Nature Park	See: study area section	0.71 (0.57–0.87)	This study
Golden jackal‐European badger	Bulgaria	District of Stara Zagora	Farmland and forest	0.59 (0.54–0.77)	Tsunoda et al. (2018)
Golden jackal‐European badger	Bulgaria	Stara Planina Mountains	Secondary oak forests	0.39 (0.34–0.46)	Tsunoda et al. (2020)
Golden jackal‐European badger	Türkiye	Sülüklü Lake Nature Park	See: study area section	0.63 (0.40–0.87)	This study
Golden jackal‐Wild boar	Thailand	Huai Kha Khaeng Wildlife Sanctuary	Dipterocarp forest, mixed deciduous	0.48 (0.45–0.49)	Charaspet et al. (2019)
Golden jackal‐Wild boar	Türkiye	Sülüklü Lake Nature Park	See: study area section	0.66 (0.42–0.81)	This study
Golden jackal‐Wildcat	Bulgaria	Stara Planina Mountains	Secondary oak forests	0.61 (0.53–0.71)	Tsunoda et al. (2020)
Golden jackal‐Wildcat	Italy	Friuli–Venezia Giulia region	Riparian lowland	0.54	Franchini et al. (2022)
Golden jackal‐Wildcat	Türkiye	Sülüklü Lake Nature Park	See: study area section	0.61 (0.41–0.81)	This study
Gray wolf‐European badger	Italy	Maremma Regional Park	Mediterranean scrub wood	0.75–0.86	Ferretti et al. (2023)
Gray wolf‐European badger	Italy	Pollino National Park	Deciduous woodland‐lowlands	0.78 (0.74–0.80)	Mori et al. (2020)
Gray wolf‐European badger	Russia	Central Forest State Nature Bio. Res.	Poor forest areas	0.63 (0.55–0.71)	Ogurtsov, Zheltukhin, and Kotlov (2018)
Gray wolf‐European badger	Italy	Maremma Regional Park	Mediterranean sclerophyll scrubland	0.85	Rossa, Lovari, and Ferretti (2021)
Gray wolf‐European badger	Türkiye	Sülüklü Lake Nature Park	See: study area section	0.36 (0.08–0.54)	This study
Gray wolf‐European hare	Italy	Pollino National Park	Deciduous woodland‐lowlands	0.88 (0.82–0.93)	Mori et al. (2020)
Gray wolf‐European hare	Türkiye	Sülüklü Lake Nature Park	See: study area section	0.45 (0.18–0.63)	This study
Gray wolf‐Martes spp.	Italy	Maremma Regional Park	Mediterranean scrub wood	0.80–0.93	Ferretti et al. (2023)
Gray wolf‐Beech marten	Türkiye	Sülüklü Lake Nature Park	See: study area section	0.48 (0.28–0.71)	This study
Gray wolf‐Roe deer	Italy	Pollino National Park	Deciduous woodland‐lowlands	0.71 (0.68–0.75)	Mori et al. (2020)
Gray wolf‐Roe deer	Italy	Maremma Regional Park	Mediterranean sclerophyll scrubland	0.61	Rossa, Lovari, and Ferretti (2021)
Gray wolf‐Roe deer	Türkiye	Sülüklü Lake Nature Park	See: study area section	0.55 (0.39–0.78)	This study
Gray wolf‐Wild boar	Italy	Pollino National Park	Deciduous woodland‐lowlands	0.88 (0.85–0.93)	Mori et al. (2020)
Gray wolf‐Wild boar	Russia	Central Forest State Nature Bio. Res.	Poor forest areas	0.78 (0.71–0.84)	Ogurtsov, Zheltukhin, and Kotlov (2018)
Gray wolf‐Wild boar	Italy	Maremma Regional Park	Mediterranean sclerophyll scrubland	0.81	Rossa, Lovari, and Ferretti (2021)
Gray wolf‐Wild boar	Türkiye	Sülüklü Lake Nature Park	See: study area section	0.55 (0.37–0.78)	This study
Gray wolf‐Wild cat	Italy	Pollino National Park	Deciduous woodland‐lowlands	0.79 (0.75–0.83)	Mori et al. (2020)
Gray wolf‐Wild cat	Türkiye	Sülüklü Lake Nature Park	See: study area section	0.62 (0.30–0.76)	This study
Red fox‐Beech marten	Mongolia	Bayan Onjuul	Steppe	0.57 (0.49–0.73)	Mori et al. (2021)
Red fox‐Beech marten	India	Lahaul and Spiti district	Dry alpine steppe	0.58	Roy et al. (2019)
Red fox‐Beech marten	Bulgaria	Stara Planina Mountains	Secondary oak forests	0.84 (0.77–0.92)	Tsunoda et al. (2020)
Red fox‐Beech marten	Türkiye	Sülüklü Lake Nature Park	See: study area section	0.60 (0.44–0.69)	This study
Red fox‐European badger	Italy	North‐eastern part of Grosseto	Deciduous woodlands	0.90	Mori and Menchetti (2019)
Red fox‐European badger	Italy	Pollino National Park	Deciduous woodland‐lowlands	0.77 (0.73–0.80)	Mori et al. (2020)
Red fox‐European badger	Bulgaria	Stara Planina Mountains	Secondary oak forests	0.74 (0.66–0.80)	Tsunoda et al. (2020)
Red fox‐European badger	Scotland	Strathspey	Seminatural forest‐forestry plantations	0.64 (0.47–0.77)	Zalewska et al. (2021)
Red fox‐European badger	Türkiye	Sülüklü Lake Nature Park	See: study area section	0.52 (0.31–0.71)	This study
Red fox‐European hare	Italy	Southern Tuscany	Deciduous woodlands	0.82 (0.74–0.90)	Viviano et al. (2021)
Red fox‐European hare	Türkiye	Sülüklü Lake Nature Park	See: study area section	0.55 (0.35 0.65)	This study
Red fox‐Golden jackal	Italy	Friuli–Venezia Giulia region	Riparian lowland	0.59	Franchini et al. (2022)
Red fox‐Golden jackal	Italy	Friuli–Venezia Giulia region	Karst plateau‐cultivated plain	0.77 (0.65–0.88)	Torretta et al. (2021)
Red fox‐Golden jackal	Bulgaria	District of Stara Zagora	Farmland and forest	0.79 (0.72–0.93)	Tsunoda et al. (2018)
Red fox‐Golden jackal	Bulgaria	Stara Planina Mountains	Secondary oak forests	0.65 (0.57–0.73)	Tsunoda et al. (2020)
Red fox‐Golden jackal	Türkiye	Sülüklü Lake Nature Park	See: study area section	0.69 (0.53–0.84)	This study
Red fox‐Gray wolf	Italy	Maremma Regional Park	Mediterranean scrub wood	0.87–0.96	Ferretti et al. (2023)
Red fox‐Gray wolf	Italy	Pollino National Park	Deciduous woodland‐lowlands	0.91 (0.86–0.96)	Mori et al. (2020)
Red fox‐Gray wolf	Italy	Maremma Regional Park	Mediterranean sclerophyll scrubland	0.88 (0.84–0.98)	Rossa, Lovari, and Ferretti (2021)
Red fox‐Gray wolf	Türkiye	Sülüklü Lake Nature Park	See: study area section	0.58 (0.38–0.87)	This study
Red fox‐Hedgehog	Italy	Venetian plain	Artificial surfaces	0.75 (0.65–0.76)	Nardotto (2022)
Red fox‐Hedgehog	Türkiye	Sülüklü Lake Nature Park	See: study area section	0.51 (0.35 0.56)	This study
Red fox–Red squirrel	Mongolia	Khangai Nuruu National Park	Forest‐alpine meadow	0.33 (0.27–0.36)	Mori et al. (2021)
Red fox–Red squirrel	Türkiye	Sülüklü Lake Nature Park	See: study area section	0.52 (0.38–0.61)	This study
Red fox‐Wild cat	Italy	Friuli–Venezia Giulia region	Pre‐Alps	0.44	Franchini et al. (2022)
Red fox‐Wild cat	Italy	Friuli–Venezia Giulia region	Riparian lowland	0.82	Franchini et al. (2022)
Red fox‐Wild cat	Italy	Pollino National Park	Deciduous woodland‐lowlands	0.81 (0.79–0.83)	Mori et al. (2020)
Red fox‐Wild cat	Bulgaria	Stara Planina Mountains	Secondary oak forests	0.88 (0.80–0.95)	Tsunoda et al. (2020)
Red fox‐Wild cat	Türkiye	Sülüklü Lake Nature Park	See: study area section	0.67 (0.54–0.83)	This study
Roe deer‐Wild boar	Italy	Pollino National Park	Deciduous woodland‐lowlands	0.78 (0.74–0.82)	Mori et al. (2020)
Roe deer‐Wild boar	Italy	The Presidential Estate of Castelporziano	Dunes, wetlands, Mediterranean scrubland	0.63 (0.53–0.73)	Zanni et al. (2021)
Roe deer‐Wild boar	Türkiye	Sülüklü Lake Nature Park	See: study area section	0.50 (0.28–0.62)	This study
Wild cat‐Beech marten	Bulgaria	Stara Planina Mountains	Secondary oak forests	0.86 (0.78–0.94)	Tsunoda et al. (2020)
Wild cat‐Beech marten	Türkiye	Sülüklü Lake Nature Park	See: study area section	0.69 (0.53–0.83)	This study
