# Supplementary material for: Daily Activity Patterns and Overlap Activity of Medium–Large Mammals in Sülüklü Lake Nature Park, Western Black Sea Region, Türkiye
Source: Ecol Evol. 2024 Dec 3;14(12):e70654. doi: 10.1002/ece3.70654 (PMC11615093; doi:10.1002/ece3.70654)
Supplement: Supplementary file 2 — Data S2. [file ECE3-14-e70654-s002.docx]

###Five important steps before running analysis

#1. Set working directory by Session --- Set working directory --- Choose directory

#2. Install packages

#3. library

#4. Import Data

#5. Run related analysis

###################Activity pattern and overlap#################

#1. Set working directory by Session --- Set working directory --- Choose directory

#2. Install packages

install.packages("overlap")

#3. library

library(overlap)

#4. Import Data

data=read.csv("veri.csv")

#5. Run related analysis

####Activity pattern######

timeRad <- data$Time * 2 * pi

VV <- timeRad[data$Species == 'Vulpes vulpes'] ##Extract Red fox data from the dataset

VV

CA<- timeRad[data$Species == 'Canis aureus'] ##Extract Golden jackal data from the dataset

CA

CC <- timeRad[data$Species == 'Capreolus capreolus'] ##Extract Roe deer data from the dataset

CC

MF <- timeRad[data$Species == 'Martes foina'] ##Extract Beech marten data from the dataset

MF

FS <- timeRad[data$Species == 'Felis silvestris'] ##Extract Wildcat data from the dataset

FS

LE <- timeRad[data$Species == 'Lepus europaeus'] ##Extract European hare data from the dataset

LE

MM <- timeRad[data$Species == 'Meles meles'] ##Extract European badger data from the dataset

MM

EC <- timeRad[data$Species == 'Erinaceus concolor'] ##Extract Southern white-breasted hedgehog data from the dataset

EC

SA <- timeRad[data$Species == 'Sciurus anomalus'] ##Extract Caucasian squirrel data from the dataset

SA

SS<- timeRad[data$Species == 'Sus scrofa'] ##Extract Wild boar data from the dataset

SS

UA <- timeRad[data$Species == 'Ursus arctos'] ##Extract Brown bear data from the dataset

UA

CL <- timeRad[data$Species == 'Canis lupus'] ##Extract Gray wolf data from the dataset

CL

LL <- timeRad[data$Species == 'Lynx lynx'] ##Extract Eurasian lynx data from the dataset

LL

#Drawing activity plot for Red fox (VV)

densityPlot(VV, rug=TRUE, adjust=1,extend=NULL,main="Red fox",ylim=c(0,0.08),ylab="Density",xlab="Time")

#Drawing activity plot for Golden jackal (CA)

densityPlot(CA, rug=TRUE, adjust=1,extend=NULL,main="Golden jackal",ylim=c(0,0.15),ylab="Density",xlab="Time")

#Drawing activity plot for Capreolus capreolus (CC)

densityPlot(CC, rug=TRUE, adjust=1,extend=NULL,main="Roe deer",ylim=c(0,0.08),ylab="Density",xlab="Time")

#Drawing activity plot for Martes foina (MF)

densityPlot(MF, rug=TRUE, adjust=1,extend=NULL,main="Beech marten",ylim=c(0,0.15),ylab="Density",xlab="Time")

#Drawing activity plot for Felis silvestris (FS)

densityPlot(FS, rug=TRUE, adjust=1,extend=NULL,main="Wildcat",ylim=c(0,0.15),ylab="Density",xlab="Time")

#Drawing activity plot for Lepus europaeus (LE)

densityPlot(LE, rug=TRUE, adjust=1,extend=NULL,main="European hare",ylim=c(0,0.15),ylab="Density",xlab="Time")

#Drawing activity plot for Meles meles (MM)

densityPlot(MM, rug=TRUE, adjust=1,extend=NULL,main="European badger",ylim=c(0,0.15),ylab="Density",xlab="Time")

#Drawing activity plot for Erinaceus concolor (EC)

densityPlot(EC, rug=TRUE, adjust=1,extend=NULL,main="Southern white-breasted hedgehog",ylim=c(0,0.15),ylab="Density",xlab="Time")

#Drawing activity plot for Sciurus anomalus (SA)

densityPlot(SA, rug=TRUE, adjust=1,extend=NULL,main="Caucasian squirrel",ylim=c(0,0.15),ylab="Density",xlab="Time")

#Drawing activity plot for Sus scrofa (SS)

densityPlot(SS, rug=TRUE, adjust=1,extend=NULL,main="Wild boar",ylim=c(0,0.15),ylab="Density",xlab="Time")

#Drawing activity plot for Ursus arctos (UA)

densityPlot(UA, rug=TRUE, adjust=1,extend=NULL,main="Brown bear",ylim=c(0,0.2),ylab="Density",xlab="Time")

#Drawing activity plot for Canis lupus (CL)

densityPlot(CL, rug=TRUE, adjust=1,extend=NULL,main="Gray wolf",ylim=c(0,0.2),ylab="Density",xlab="Time")

#Drawing activity plot for Lynx lynx (LL)

densityPlot(LL, rug=TRUE, adjust=1,extend=NULL,main="Eurasian lynx",ylim=c(0,0.2),ylab="Density",xlab="Time")

###Activity Overlap######

### Overlap between Red fox (VV) and Golden jackal (CA)

min(length(VV), length(CA)) #Checking minimum length from both species

rVV.CA <- overlapEst(VV,CA) ###Overlap for both species by using overlapEst function

rVV.CA

#b1 (Dhat1) for lower than 75 observation

#b4 (Dhat4) for higher than 75 observation

#####Drawing overlap plot VV vs CA

overlapPlot(VV,CA,main="", ylim=c(0,0.15),cex.axis=1.5,cex.lab=1.5,cex.main=1.5, xlab = "", ylab = "")

legend('topleft', c("Red Fox", "Golden Jackal"), lty=c(1,2), col=c(1,4), bty='n',cex=1.5)

text(x = 21, y = 0.145, labels = sprintf("Dhat1: 0.69"), col = "black", cex = 1.3)

par(mar = c(3, 3, 2, 1))

###To estimate confidence intervals we need to know the sampling distribution

###which our coefficient of overlapping is drawn from,

###i.e, the distribution we would get if we had a very large number of independent samples from nature.

###The best way to investigate this is to use a bootstrap.

VVboot<-resample(VV,1000) ###2000 times Bootstrap for Red fox (VV) by using resample function

CAboot<-resample(CA,1000) ###2000 times Bootstrap for Golden jackal (CA) by using resample function

VV.CAboot <- bootEst(VVboot, CAboot) ###Bootstrap estimate for both VVboot and CAboot

BSVV.CA<-colMeans(VV.CAboot) ###Mean of Bootstrap value

BSVV.CA

VV.CAM<-VV.CAboot[,2] #Extract the require column of the matrix

bootCI(rVV.CA[2],VV.CAM)

###Activity Overlap######

### Overlap between Red fox (VV) and Roe deer (CC)

min(length(VV), length(CC)) #Checking minimum length from both species

rVV.CC <- overlapEst(VV,CC) ###Overlap for both species by using overlapEst function

rVV.CC

#b1 (Dhat1) for lower than 75 observation

#b4 (Dhat4) for higher than 75 observation

#####Drawing overlap plot VV vs CC

overlapPlot(VV,CC, main="",ylim=c(0,0.1),cex.axis=1.5,cex.lab=1.5,cex.main=1.5, xlab = "", ylab = "")

legend('topleft', c("Red Fox", "Roe Deer"), lty=c(1,2), col=c(1,4), bty='n',cex=1.5)

text(x = 21, y = 0.099, labels = sprintf("Dhat1: 0.77"), col = "black", cex = 1.3)

par(mar = c(3, 3, 2, 1))

###To estimate confidence intervals we need to know the sampling distribution

###which our coefficient of overlapping is drawn from,

###i.e, the distribution we would get if we had a very large number of independent samples from nature.

###The best way to investigate this is to use a bootstrap.

VVboot<-resample(VV,1000) ###2000 times Bootstrap for Red fox (VV) by using resample function

CCboot<-resample(CC,1000) ###2000 times Bootstrap for Roe deer (CC) by using resample function

VV.CCboot <- bootEst(VVboot, CCboot) ###Bootstrap estimate for both VVboot and CCboot

BSVV.CC<-colMeans(VV.CCboot) ###Mean of Bootstrap value

BSVV.CC

VV.CAM<-VV.CCboot[,2] #Extract the require column of the matrix

bootCI(rVV.CC[2],VV.CAM)

###Activity Overlap######

### Overlap between Red fox (VV) and Beech marten (MF)

min(length(VV), length(MF)) #Checking minimum length from both species

rVV.MF <- overlapEst(VV,MF) ###Overlap for both species by using overlapEst function

rVV.MF

#b1 (Dhat1) for lower than 75 observation

#b4 (Dhat4) for higher than 75 observation

#####Drawing overlap plot VV vs MF

overlapPlot(VV,MF, main="",ylim=c(0,0.15),cex.axis=1.5,cex.lab=1.5,cex.main=1.5, xlab = "", ylab = "")

legend('topleft', c("Red Fox", "Beech Marten"), lty=c(1,2), col=c(1,4), bty='n',cex=1.5)

text(x = 21.5, y = 0.145, labels = sprintf("Dhat4: 0.59"), col = "black", cex = 1.3)

par(mar = c(3, 3, 2, 1))

###To estimate confidence intervals we need to know the sampling distribution

###which our coefficient of overlapping is drawn from,

###i.e, the distribution we would get if we had a very large number of independent samples from nature.

###The best way to investigate this is to use a bootstrap.

VVboot<-resample(VV,1000) ###2000 times Bootstrap for Red fox (VV) by using resample function

MFboot<-resample(MF,1000) ###2000 times Bootstrap for Beech marten (CC) by using resample function

VV.MFboot <- bootEst(VVboot, MFboot) ###Bootstrap estimate for both VVboot and MFboot

BSVV.MF<-colMeans(VV.MFboot) ###Mean of Bootstrap value

BSVV.MF

VV.CAM<-VV.MFboot[,2] #Extract the require column of the matrix

bootCI(rVV.MF[2],VV.CAM)

###Activity Overlap######

### Overlap between Red fox (VV) and Felis silvestris (FS)

min(length(VV), length(FS)) #Checking minimum length from both species

rVV.FS <- overlapEst(VV,FS) ###Overlap for both species by using overlapEst function

rVV.FS

#b1 (Dhat1) for lower than 75 observation

#b4 (Dhat4) for higher than 75 observation

#####Drawing overlap plot VV vs FS

overlapPlot(VV,FS, main="",ylim=c(0,0.15),cex.axis=1.5,cex.lab=1.5,cex.main=1.5, xlab = "", ylab = "")

legend('topleft', c("Red Fox", "Wild cat"), lty=c(1,2), col=c(1,4), bty='n',cex=1.5)

text(x = 21, y = 0.145, labels = sprintf("Dhat1: 0.67"), col = "black", cex = 1.3)

par(mar = c(3, 3, 2, 1))

###To estimate confidence intervals we need to know the sampling distribution

###which our coefficient of overlapping is drawn from,

###i.e, the distribution we would get if we had a very large number of independent samples from nature.

###The best way to investigate this is to use a bootstrap.

VVboot<-resample(VV,1000) ###2000 times Bootstrap for Red fox (VV) by using resample function

FSboot<-resample(FS,1000) ###2000 times Bootstrap for Wild cat (FS) by using resample function

VV.FSboot <- bootEst(VVboot, FSboot) ###Bootstrap estimate for both VVboot and FSboot

BSVV.FS<-colMeans(VV.FSboot) ###Mean of Bootstrap value

BSVV.FS

VV.CAM<-VV.FSboot[,2] #Extract the require column of the matrix

bootCI(rVV.FS[2],VV.CAM)

###Activity Overlap######

### Overlap between Red fox (VV) and European hare (LE)

min(length(VV), length(LE)) #Checking minimum length from both species

rVV.LE <- overlapEst(VV,LE) ###Overlap for both species by using overlapEst function

rVV.LE

#b1 (Dhat1) for lower than 75 observation

#b4 (Dhat4) for higher than 75 observation

#####Drawing overlap plot VV vs LE

overlapPlot(VV,LE, main="",ylim=c(0,0.15),cex.axis=1.5,cex.lab=1.5,cex.main=1.5, xlab = "", ylab = "")

legend('topleft', c("Red Fox", "European Hare"), lty=c(1,2), col=c(1,4), bty='n',cex=1.5)

text(x = 21, y = 0.145, labels = sprintf("Dhat1: 0.55"), col = "black", cex = 1.3)

par(mar = c(3, 3, 2, 1))

###To estimate confidence intervals we need to know the sampling distribution

###which our coefficient of overlapping is drawn from,

###i.e, the distribution we would get if we had a very large number of independent samples from nature.

###The best way to investigate this is to use a bootstrap.

VVboot<-resample(VV,1000) ###2000 times Bootstrap for Red fox (VV) by using resample function

LEboot<-resample(LE,1000) ###2000 times Bootstrap for European hare (LE) by using resample function

VV.LEboot <- bootEst(VVboot, LEboot) ###Bootstrap estimate for both VVboot and LEboot

BSVV.LE<-colMeans(VV.LEboot) ###Mean of Bootstrap value

BSVV.LE

VV.CAM<-VV.LEboot[,2] #Extract the require column of the matrix

bootCI(rVV.LE[2],VV.CAM)

###Activity Overlap######

### Overlap between Red fox (VV) and European badger (MM)

min(length(VV), length(MM)) #Checking minimum length from both species

rVV.MM <- overlapEst(VV,MM) ###Overlap for both species by using overlapEst function

rVV.MM

#b1 (Dhat1) for lower than 75 observation

#b4 (Dhat4) for higher than 75 observation

#####Drawing overlap plot VV vs MM

overlapPlot(VV,MM, main="",ylim=c(0,0.15),cex.axis=1.5,cex.lab=1.5,cex.main=1.5, xlab = "", ylab = "")

legend('topleft', c("Red Fox", "European Badger"), lty=c(1,2), col=c(1,4), bty='n',cex=1.5)

text(x = 21, y = 0.145, labels = sprintf("Dhat1: 0.52"), col = "black", cex = 1.3)

par(mar = c(3, 3, 2, 1))

###To estimate confidence intervals we need to know the sampling distribution

###which our coefficient of overlapping is drawn from,

###i.e, the distribution we would get if we had a very large number of independent samples from nature.

###The best way to investigate this is to use a bootstrap.

VVboot<-resample(VV,1000) ###2000 times Bootstrap for Red fox (VV) by using resample function

MMboot<-resample(MM,1000) ###2000 times Bootstrap for European badger (MM) by using resample function

VV.MMboot <- bootEst(VVboot, MMboot) ###Bootstrap estimate for both VVboot and MMboot

BSVV.MM<-colMeans(VV.MMboot) ###Mean of Bootstrap value

BSVV.MM

VV.CAM<-VV.MMboot[,2] #Extract the require column of the matrix

bootCI(rVV.MM[2],VV.CAM)

###Activity Overlap######

### Overlap between Red fox (VV) and Southern white-breasted hedgehog (EC)

min(length(VV), length(EC)) #Checking minimum length from both species

rVV.EC <- overlapEst(VV,EC) ###Overlap for both species by using overlapEst function

rVV.EC

#b1 (Dhat1) for lower than 75 observation

#b4 (Dhat4) for higher than 75 observation

#####Drawing overlap plot VV vs EC

overlapPlot(VV,EC, main="",ylim=c(0,0.15),cex.axis=1.5,cex.lab=1.5,cex.main=1.5, xlab = "", ylab = "")

legend('topleft', c("Red Fox", "Hedgehog"), lty=c(1,2), col=c(1,4), bty='n',cex=1.5)

text(x = 21, y = 0.145, labels = sprintf("Dhat4: 0.50"), col = "black", cex = 1.3)

par(mar = c(3, 3, 2, 1))

###To estimate confidence intervals we need to know the sampling distribution

###which our coefficient of overlapping is drawn from,

###i.e, the distribution we would get if we had a very large number of independent samples from nature.

###The best way to investigate this is to use a bootstrap.

VVboot<-resample(VV,1000) ###2000 times Bootstrap for Red fox (VV) by using resample function

ECboot<-resample(EC,1000) ###2000 times Bootstrap for Southern white-breasted hedgehog (EC) by using resample function

VV.ECboot <- bootEst(VVboot, ECboot) ###Bootstrap estimate for both VVboot and ECboot

BSVV.EC<-colMeans(VV.ECboot) ###Mean of Bootstrap value

BSVV.EC

VV.CAM<-VV.ECboot[,2] #Extract the require column of the matrix

bootCI(rVV.EC[2],VV.CAM)

###Activity Overlap######

### Overlap between Red fox (VV) and Caucasian squirrel (SA)

min(length(VV), length(SA)) #Checking minimum length from both species

rVV.SA <- overlapEst(VV,SA) ###Overlap for both species by using overlapEst function

rVV.SA

#b1 (Dhat1) for lower than 75 observation

#b4 (Dhat4) for higher than 75 observation

#####Drawing overlap plot VV vs SA

overlapPlot(VV,SA, main="",ylim=c(0,0.15),cex.axis=1.5,cex.lab=1.5,cex.main=1.5, xlab = "", ylab = "")

legend('topleft', c("Red Fox", "Caucasian Squirrel"), lty=c(1,2), col=c(1,4), bty='n',cex=1.5)

text(x = 21, y = 0.145, labels = sprintf("Dhat1: 0.52"), col = "black", cex = 1.3)

par(mar = c(3, 3, 2, 1))

###To estimate confidence intervals we need to know the sampling distribution

###which our coefficient of overlapping is drawn from,

###i.e, the distribution we would get if we had a very large number of independent samples from nature.

###The best way to investigate this is to use a bootstrap.

VVboot<-resample(VV,1000) ###2000 times Bootstrap for Red fox (VV) by using resample function

SAboot<-resample(SA,1000) ###2000 times Bootstrap for Caucasian squirrel (SA) by using resample function

VV.SAboot <- bootEst(VVboot, SAboot) ###Bootstrap estimate for both VVboot and SAboot

BSVV.SA<-colMeans(VV.SAboot) ###Mean of Bootstrap value

BSVV.SA

VV.CAM<-VV.SAboot[,2] #Extract the require column of the matrix

bootCI(rVV.SA[2],VV.CAM)

###Activity Overlap######

### Overlap between Red fox (VV) and Wild boar (SS)

min(length(VV), length(SS)) #Checking minimum length from both species

rVV.SS <- overlapEst(VV,SS) ###Overlap for both species by using overlapEst function

rVV.SS

#b1 (Dhat1) for lower than 75 observation

#b4 (Dhat4) for higher than 75 observation

#####Drawing overlap plot VV vs SS

overlapPlot(VV,SS, main="",ylim=c(0,0.15),cex.axis=1.5,cex.lab=1.5,cex.main=1.5, xlab = "", ylab = "")

legend('topleft', c("Red Fox", "Wild Boar"), lty=c(1,2), col=c(1,4), bty='n',cex=1.5)

text(x = 21, y = 0.145, labels = sprintf("Dhat1: 0.57"), col = "black", cex = 1.3)

par(mar = c(3, 3, 2, 1))

###To estimate confidence intervals we need to know the sampling distribution

###which our coefficient of overlapping is drawn from,

###i.e, the distribution we would get if we had a very large number of independent samples from nature.

###The best way to investigate this is to use a bootstrap.

VVboot<-resample(VV,1000) ###2000 times Bootstrap for Red fox (VV) by using resample function

SSboot<-resample(SS,1000) ###2000 times Bootstrap for Wild boar (SS) by using resample function

VV.SSboot <- bootEst(VVboot, SSboot) ###Bootstrap estimate for both VVboot and SSboot

BSVV.SS<-colMeans(VV.SSboot) ###Mean of Bootstrap value

BSVV.SS

VV.CAM<-VV.SSboot[,2] #Extract the require column of the matrix

bootCI(rVV.SS[2],VV.CAM)

###Activity Overlap######

### Overlap between Red fox (VV) and Gray wolf (CL)

min(length(VV), length(CL)) #Checking minimum length from both species

rVV.CL <- overlapEst(VV,CL) ###Overlap for both species by using overlapEst function

rVV.CL

#b1 (Dhat1) for lower than 75 observation

#b4 (Dhat4) for higher than 75 observation

#####Drawing overlap plot VV vs CL

overlapPlot(VV,CL, main="",ylim=c(0,0.15),cex.axis=1.5,cex.lab=1.5,cex.main=1.5, xlab = "", ylab = "")

legend('topleft', c("Red Fox", "Gray Wolf"), lty=c(1,2), col=c(1,4), bty='n',cex=1.5)

text(x = 21, y = 0.145, labels = sprintf("Dhat1: 0.58"), col = "black", cex = 1.3)

par(mar = c(3, 3, 2, 1))

###To estimate confidence intervals we need to know the sampling distribution

###which our coefficient of overlapping is drawn from,

###i.e, the distribution we would get if we had a very large number of independent samples from nature.

###The best way to investigate this is to use a bootstrap.

VVboot<-resample(VV,1000) ###2000 times Bootstrap for Red fox (VV) by using resample function

CLboot<-resample(CL,1000) ###2000 times Bootstrap for Gray wolf (CL) by using resample function

VV.CLboot <- bootEst(VVboot, CLboot) ###Bootstrap estimate for both VVboot and CLboot

BSVV.CL<-colMeans(VV.CLboot) ###Mean of Bootstrap value

BSVV.CL

VV.CAM<-VV.CLboot[,2] #Extract the require column of the matrix

bootCI(rVV.CL[2],VV.CAM)

###Activity Overlap######

### Overlap between Golden jackal (CA) and Gray wolf (CL)

min(length(CA), length(CL)) #Checking minimum length from both species

rCA.CL <- overlapEst(CA,CL) ###Overlap for both species by using overlapEst function

rCA.CL

#b1 (Dhat1) for lower than 75 observation

#b4 (Dhat4) for higher than 75 observation

#####Drawing overlap plot CA vs CL

overlapPlot(CA,CL, main="" , ylim=c(0,0.15),cex.axis=1.5,cex.lab=1.5,cex.main=1.5, xlab = "", ylab = "")

legend('topleft', c("Golden jackal", "Gray Wolf"), lty=c(1,2), col=c(1,4), bty='n',cex=1.5)

text(x = 21, y = 0.145, labels = sprintf("Dhat1: 0.49"), col = "black", cex = 1.3)

par(mar = c(3, 3, 2, 1))

###To estimate confidence intervals we need to know the sampling distribution

###which our coefficient of overlapping is drawn from,

###i.e, the distribution we would get if we had a very large number of independent samples from nature.

###The best way to investigate this is to use a bootstrap.

CAboot<-resample(CA,1000) ###2000 times Bootstrap for Golden jackal (CA) by using resample function

CLboot<-resample(CL,1000) ###2000 times Bootstrap for Gray wolf (CL) by using resample function

CA.CLboot <- bootEst(CAboot, CLboot) ###Bootstrap estimate for both CAboot and CLboot

BSCA.CL<-colMeans(CA.CLboot) ###Mean of Bootstrap value

BSCA.CL

CA.CAM<-CA.CLboot[,2] #Extract the require column of the matrix

bootCI(rCA.CL[2],CA.CAM)

###Activity Overlap######

### Overlap between Roe deer (CC) and Gray wolf (CL)

min(length(CC), length(CL)) #Checking minimum length from both species

rCC.CL <- overlapEst(CC,CL) ###Overlap for both species by using overlapEst function

rCC.CL

#b1 (Dhat1) for lower than 75 observation

#b4 (Dhat4) for higher than 75 observation

#####Drawing overlap plot CC vs CL

overlapPlot(CC,CL, main="",ylim=c(0,0.15),cex.axis=1.5,cex.lab=1.5,cex.main=1.5, xlab = "", ylab = "")

legend('topleft', c("Roe deer", "Gray Wolf"), lty=c(1,2), col=c(1,4), bty='n',cex=1.5)

text(x = 21, y = 0.145, labels = sprintf("Dhat1: 0.55"), col = "black", cex = 1.3)

par(mar = c(3, 3, 2, 1))

###To estimate confidence intervals we need to know the sampling distribution

###which our coefficient of overlapping is drawn from,

###i.e, the distribution we would get if we had a very large number of independent samples from nature.

###The best way to investigate this is to use a bootstrap.

CCboot<-resample(CC,1000) ###2000 times Bootstrap for Roe deer (CC) by using resample function

CLboot<-resample(CL,1000) ###2000 times Bootstrap for Gray wolf (CL) by using resample function

CC.CLboot <- bootEst(CCboot, CLboot) ###Bootstrap estimate for both CCboot and CLboot

BSCC.CL<-colMeans(CC.CLboot) ###Mean of Bootstrap value

BSCC.CL

CC.CAM<-CC.CLboot[,2] #Extract the require column of the matrix

bootCI(rCC.CL[2],CC.CAM)

###Activity Overlap######

### Overlap between Southern white-breasted hedgehog (EC) and Gray wolf (CL)

min(length(EC), length(CL)) #Checking minimum length from both species

rEC.CL <- overlapEst(EC,CL) ###Overlap for both species by using overlapEst function

rEC.CL

#b1 (Dhat1) for lower than 75 observation

#b4 (Dhat4) for higher than 75 observation

#####Drawing overlap plot EC vs CL

overlapPlot(EC,CL, main="",ylim=c(0,0.15),cex.axis=1.5,cex.lab=1.5,cex.main=1.5, xlab = "", ylab = "")

legend('topleft', c("Hedgehog", "Gray Wolf"), lty=c(1,2), col=c(1,4), bty='n',cex=1.5)

text(x = 21, y = 0.145, labels = sprintf("Dhat1: 0.40"), col = "black", cex = 1.3)

par(mar = c(3, 3, 2, 1))

###To estimate confidence intervals we need to know the sampling distribution

###which our coefficient of overlapping is drawn from,

###i.e, the distribution we would get if we had a very large number of independent samples from nature.

###The best way to investigate this is to use a bootstrap.

ECboot<-resample(EC,1000) ###2000 times Bootstrap for Southern white-breasted hedgehog (EC) by using resample function

CLboot<-resample(CL,1000) ###2000 times Bootstrap for Gray Wolf (CL) by using resample function

EC.CLboot <- bootEst(ECboot, CLboot) ###Bootstrap estimate for both ECboot and CLboot

BSEC.CL<-colMeans(EC.CLboot) ###Mean of Bootstrap value

BSEC.CL

EC.CAM<-EC.CLboot[,2] #Extract the require column of the matrix

bootCI(rEC.CL[2],EC.CAM)

###Activity Overlap######

### Overlap between Beech marten (MF) and Gray wolf (CL)

min(length(MF), length(CL)) #Checking minimum length from both species

rMF.CL <- overlapEst(MF,CL) ###Overlap for both species by using overlapEst function

rMF.CL

#b1 (Dhat1) for lower than 75 observation

#b4 (Dhat4) for higher than 75 observation

#####Drawing overlap plot MF vs CL

overlapPlot(MF,CL, main="",ylim=c(0,0.15),cex.axis=1.5,cex.lab=1.5,cex.main=1.5, xlab = "", ylab = "")

legend('topleft', c("Beech marten", "Gray Wolf"), lty=c(1,2), col=c(1,4), bty='n',cex=1.5)

text(x = 21, y = 0.145, labels = sprintf("Dhat1: 0.48"), col = "black", cex = 1.3)

par(mar = c(3, 3, 2, 1))

###To estimate confidence intervals we need to know the sampling distribution

###which our coefficient of overlapping is drawn from,

###i.e, the distribution we would get if we had a very large number of independent samples from nature.

###The best way to investigate this is to use a bootstrap.

MFboot<-resample(MF,1000) ###2000 times Bootstrap for Beech marten (MF) by using resample function

CLboot<-resample(CL,1000) ###2000 times Bootstrap for Gray Wolf (CL) by using resample function

MF.CLboot <- bootEst(MFboot, CLboot) ###Bootstrap estimate for both MFboot and CLboot

BSMF.CL<-colMeans(MF.CLboot) ###Mean of Bootstrap value

BSMF.CL

MF.CAM<-MF.CLboot[,2] #Extract the require column of the matrix

bootCI(rMF.CL[2],MF.CAM)

###Activity Overlap######

### Overlap between Caucasian squirrel (SA) and Gray wolf (CL)

min(length(SA), length(CL)) #Checking minimum length from both species

rSA.CL <- overlapEst(SA,CL) ###Overlap for both species by using overlapEst function

rSA.CL

#b1 (Dhat1) for lower than 75 observation

#b4 (Dhat4) for higher than 75 observation

#####Drawing overlap plot SA vs CL

overlapPlot(SA,CL, main="",ylim=c(0,0.15),cex.axis=1.5,cex.lab=1.5,cex.main=1.5, xlab = "", ylab = "")

legend('topleft', c("Caucasian squirrel", "Gray wolf"), lty=c(1,2), col=c(1,4), bty='n',cex=1.5)

text(x = 21, y = 0.145, labels = sprintf("Dhat1: 0.62"), col = "black", cex = 1.3)

par(mar = c(3, 3, 2, 1))

###To estimate confidence intervals we need to know the sampling distribution

###which our coefficient of overlapping is drawn from,

###i.e, the distribution we would get if we had a very large number of independent samples from nature.

###The best way to investigate this is to use a bootstrap.

SAboot<-resample(SA,1000) ###2000 times Bootstrap for Caucasian squirrel (SA) by using resample function

SAboot<-resample(CL,1000) ###2000 times Bootstrap for Gray Wolf (CL) by using resample function

SA.CLboot <- bootEst(SAboot, CLboot) ###Bootstrap estimate for both SAboot and CLboot

BSSA.CL<-colMeans(SA.CLboot) ###Mean of Bootstrap value

BSSA.CL

SA.CAM<-SA.CLboot[,2] #Extract the require column of the matrix

bootCI(rSA.CL[2],SA.CAM)

###Activity Overlap######

### Overlap between Wildcat (FS) and Gray wolf (CL)

min(length(FS), length(CL)) #Checking minimum length from both species

rFS.CL <- overlapEst(FS,CL) ###Overlap for both species by using overlapEst function

rFS.CL

#b1 (Dhat1) for lower than 75 observation

#b4 (Dhat4) for higher than 75 observation

#####Drawing overlap plot FS vs CL

overlapPlot(FS,CL, main="",ylim=c(0,0.15),cex.axis=1.5,cex.lab=1.5,cex.main=1.5, xlab = "", ylab = "")

legend('topleft', c("Wildcat", "Gray wolf"), lty=c(1,2), col=c(1,4), bty='n',cex=1.5)

text(x = 21, y = 0.145, labels = sprintf("Dhat1: 0.62"), col = "black", cex = 1.3)

par(mar = c(3, 3, 2, 1))

###To estimate confidence intervals we need to know the sampling distribution

###which our coefficient of overlapping is drawn from,

###i.e, the distribution we would get if we had a very large number of independent samples from nature.

###The best way to investigate this is to use a bootstrap.

FSboot<-resample(FS,1000) ###2000 times Bootstrap for Wildcat (FS) by using resample function

FSboot<-resample(CL,1000) ###2000 times Bootstrap for Gray Wolf (CL) by using resample function

FS.CLboot <- bootEst(FSboot, CLboot) ###Bootstrap estimate for both FSboot and CLboot

BSFS.CL<-colMeans(FS.CLboot) ###Mean of Bootstrap value

BSFS.CL

FS.CAM<-FS.CLboot[,2] #Extract the require column of the matrix

bootCI(rFS.CL[2],FS.CAM)

###Activity Overlap######

### Overlap between European hare (LE) and Gray wolf (CL)

min(length(LE), length(CL)) #Checking minimum length from both species

rLE.CL <- overlapEst(LE,CL) ###Overlap for both species by using overlapEst function

rLE.CL

#b1 (Dhat1) for lower than 75 observation

#b4 (Dhat4) for higher than 75 observation

#####Drawing overlap plot LE vs CL

overlapPlot(LE,CL, main="",ylim=c(0,0.15),cex.axis=1.5,cex.lab=1.5,cex.main=1.5, xlab = "", ylab = "")

legend('topleft', c("European hare", "Gray wolf"), lty=c(1,2), col=c(1,4), bty='n',cex=1.5)

text(x = 21, y = 0.145, labels = sprintf("Dhat1: 0.45"), col = "black", cex = 1.3)

par(mar = c(3, 3, 2, 1))

###To estimate confidence intervals we need to know the sampling distribution

###which our coefficient of overlapping is drawn from,

###i.e, the distribution we would get if we had a very large number of independent samples from nature.

###The best way to investigate this is to use a bootstrap.

LEboot<-resample(LE,1000) ###2000 times Bootstrap for European hare (LE) by using resample function

CLboot<-resample(CL,1000) ###2000 times Bootstrap for Gray Wolf (CL) by using resample function

LE.CLboot <- bootEst(LEboot, CLboot) ###Bootstrap estimate for both LEboot and CLboot

BSLE.CL<-colMeans(LE.CLboot) ###Mean of Bootstrap value

BSLE.CL

LE.CAM<-LE.CLboot[,2] #Extract the require column of the matrix

bootCI(rLE.CL[2],LE.CAM)

###Activity Overlap######

### Overlap between European badger (MM) and Gray wolf (CL)

min(length(MM), length(CL)) #Checking minimum length from both species

rMM.CL <- overlapEst(MM,CL) ###Overlap for both species by using overlapEst function

rMM.CL

#b1 (Dhat1) for lower than 75 observation

#b4 (Dhat4) for higher than 75 observation

#####Drawing overlap plot MM vs CL

overlapPlot(MM,CL, main="",ylim=c(0,0.15),cex.axis=1.5,cex.lab=1.5,cex.main=1.5, xlab = "", ylab = "")

legend('topleft', c("European badger", "Gray wolf"), lty=c(1,2), col=c(1,4), bty='n',cex=1.5)

text(x = 21, y = 0.145, labels = sprintf("Dhat1: 0.36"), col = "black", cex = 1.3)

par(mar = c(3, 3, 2, 1))

###To estimate confidence intervals we need to know the sampling distribution

###which our coefficient of overlapping is drawn from,

###i.e, the distribution we would get if we had a very large number of independent samples from nature.

###The best way to investigate this is to use a bootstrap.

MMboot<-resample(MM,1000) ###2000 times Bootstrap for European badger (MM) by using resample function

CLboot<-resample(CL,1000) ###2000 times Bootstrap for Gray Wolf (CL) by using resample function

MM.CLboot <- bootEst(MMboot, CLboot) ###Bootstrap estimate for both MMboot and CLboot

BSMM.CL<-colMeans(MM.CLboot) ###Mean of Bootstrap value

BSMM.CL

MM.CAM<-MM.CLboot[,2] #Extract the require column of the matrix

bootCI(rMM.CL[2],MM.CAM)

###Activity Overlap######

### Overlap between Brown bear (UA) and Gray wolf (CL)

min(length(UA), length(CL)) #Checking minimum length from both species

rUA.CL <- overlapEst(UA,CL) ###Overlap for both species by using overlapEst function

rUA.CL

#b1 (Dhat1) for lower than 75 observation

#b4 (Dhat4) for higher than 75 observation

#####Drawing overlap plot UA vs CL

overlapPlot(UA,CL, main="",ylim=c(0,0.20),cex.axis=1.5,cex.lab=1.5,cex.main=1.5, xlab = "", ylab = "")

legend('topleft', c("Brown bear", "Gray wolf"), lty=c(1,2), col=c(1,4), bty='n',cex=1.5)

text(x = 22, y = 0.08, labels = sprintf("Dhat1: 0.77"), col = "black", cex = 1.2)

par(mar = c(3, 3, 2, 2))

###To estimate confidence intervals we need to know the sampling distribution

###which our coefficient of overlapping is drawn from,

###i.e, the distribution we would get if we had a very large number of independent samples from nature.

###The best way to investigate this is to use a bootstrap.

UAboot<-resample(UA,1000) ###2000 times Bootstrap for Brown bear (UA) by using resample function

CLboot<-resample(CL,1000) ###2000 times Bootstrap for Gray Wolf (CL) by using resample function

UA.CLboot <- bootEst(UAboot, CLboot) ###Bootstrap estimate for both UAboot and CLboot

BSUA.CL<-colMeans(UA.CLboot) ###Mean of Bootstrap value

BSUA.CL

UA.CAM<-UA.CLboot[,2] #Extract the require column of the matrix

bootCI(rUA.CL[2],UA.CAM)

###Activity Overlap######

### Overlap between Wild boar (SS) and Gray wolf (CL)

min(length(SS), length(CL)) #Checking minimum length from both species

rSS.CL <- overlapEst(SS,CL) ###Overlap for both species by using overlapEst function

rSS.CL

#b1 (Dhat1) for lower than 75 observation

#b4 (Dhat4) for higher than 75 observation

#####Drawing overlap plot SS vs CL

overlapPlot(SS,CL, main="",ylim=c(0,0.20),cex.axis=1.5,cex.lab=1.5,cex.main=1.5, xlab = "", ylab = "")

legend('topleft', c("Wild boar", "Gray wolf"), lty=c(1,2), col=c(1,4), bty='n',cex=1.5)

text(x = 21, y = 0.19, labels = sprintf("Dhat1: 0.55"), col = "black", cex = 1.3)

par(mar = c(3, 3, 2, 1))

###To estimate confidence intervals we need to know the sampling distribution

###which our coefficient of overlapping is drawn from,

###i.e, the distribution we would get if we had a very large number of independent samples from nature.

###The best way to investigate this is to use a bootstrap.

SSboot<-resample(SS,1000) ###2000 times Bootstrap for Wild boar (SS) by using resample function

CLboot<-resample(CL,1000) ###2000 times Bootstrap for Gray Wolf (CL) by using resample function

SS.CLboot <- bootEst(SSboot, CLboot) ###Bootstrap estimate for both SSboot and CLboot

BSSS.CL<-colMeans(SS.CLboot) ###Mean of Bootstrap value

BSSS.CL

SS.CAM<-SS.CLboot[,2] #Extract the require column of the matrix

bootCI(rSS.CL[2],SS.CAM)

###Activity Overlap######

### Overlap between Golden jackal (CA) and Roe deer (CC)

min(length(CA), length(CC)) #Checking minimum length from both species

rCA.CL <- overlapEst(CA,CC) ###Overlap for both species by using overlapEst function

rCA.CL

#b1 (Dhat1) for lower than 75 observation

#b4 (Dhat4) for higher than 75 observation

#####Drawing overlap plot CA vs CC

overlapPlot(CA,CC, main="",ylim=c(0,0.15),cex.axis=1.5,cex.lab=1.5,cex.main=1.5, xlab = "", ylab = "")

legend('topleft', c("Golden jackal", "Roe deer"), lty=c(1,2), col=c(1,4), bty='n',cex=1.5)

text(x = 21, y = 0.145, labels = sprintf("Dhat1: 0.62"), col = "black", cex = 1.3)

par(mar = c(3, 3, 2, 1))

###To estimate confidence intervals we need to know the sampling distribution

###which our coefficient of overlapping is drawn from,

###i.e, the distribution we would get if we had a very large number of independent samples from nature.

###The best way to investigate this is to use a bootstrap.

CAboot<-resample(CA,1000) ###2000 times Bootstrap for Golden jackal (CA) by using resample function

CCboot<-resample(CC,1000) ###2000 times Bootstrap for Roe deer (CC) by using resample function

CA.CCboot <- bootEst(CAboot, CCboot) ###Bootstrap estimate for both CAboot and CCboot

BSCA.CC<-colMeans(CA.CCboot) ###Mean of Bootstrap value

BSCA.CC

CA.CAM<-CA.CCboot[,2] #Extract the require column of the matrix

bootCI(rCA.CC[2],CA.CAM)

###Activity Overlap######

### Overlap between Golden jackal (CA) and Southern white-breasted hedgehog (EC)

min(length(CA), length(EC)) #Checking minimum length from both species

rCA.EC <- overlapEst(CA,EC) ###Overlap for both species by using overlapEst function

rCA.EC

#b1 (Dhat1) for lower than 75 observation

#b4 (Dhat4) for higher than 75 observation

#####Drawing overlap plot CA vs EC

overlapPlot(CA,EC, main="",ylim=c(0,0.15),cex.axis=1.5,cex.lab=1.5,cex.main=1.5, xlab = "", ylab = "")

legend('topleft', c("Golden jackal", "Hedgehog"), lty=c(1,2), col=c(1,4), bty='n',cex=1.5)

text(x = 21, y = 0.145, labels = sprintf("Dhat1: 0.65"), col = "black", cex = 1.3)

par(mar = c(3, 3, 2, 1))

###To estimate confidence intervals we need to know the sampling distribution

###which our coefficient of overlapping is drawn from,

###i.e, the distribution we would get if we had a very large number of independent samples from nature.

###The best way to investigate this is to use a bootstrap.

CAboot<-resample(CA,1000) ###2000 times Bootstrap for Golden jackal (CA) by using resample function

ECboot<-resample(EC,1000) ###2000 times Bootstrap for Southern white-breasted hedgehog (EC) by using resample function

CA.ECboot <- bootEst(CAboot, ECboot) ###Bootstrap estimate for both CAboot and ECboot

BSCA.EC<-colMeans(CA.ECboot) ###Mean of Bootstrap value

BSCA.EC

CA.CAM<-CA.ECboot[,2] #Extract the require column of the matrix

bootCI(rCA.EC[2],CA.CAM)

###Activity Overlap######

### Overlap between Golden jackal (CA) and Beech marten (MF)

min(length(CA), length(MF)) #Checking minimum length from both species

rCA.MF <- overlapEst(CA,MF) ###Overlap for both species by using overlapEst function

rCA.MF

#b1 (Dhat1) for lower than 75 observation

#b4 (Dhat4) for higher than 75 observation

#####Drawing overlap plot CA vs MF

overlapPlot(CA,MF, main="",ylim=c(0,0.15),cex.axis=1.5,cex.lab=1.5,cex.main=1.5, xlab = "", ylab = "")

legend('topleft', c("Golden jackal", "Beech marten"), lty=c(1,2), col=c(1,4), bty='n',cex=1.5)

text(x = 21, y = 0.145, labels = sprintf("Dhat1: 0.71"), col = "black", cex = 1.2)

par(mar = c(3, 3, 2, 1))

###To estimate confidence intervals we need to know the sampling distribution

###which our coefficient of overlapping is drawn from,

###i.e, the distribution we would get if we had a very large number of independent samples from nature.

###The best way to investigate this is to use a bootstrap.

CAboot<-resample(CA,1000) ###2000 times Bootstrap for Golden jackal (CA) by using resample function

MFboot<-resample(MF,1000) ###2000 times Bootstrap for Beech marten (MF) by using resample function

CA.MFboot <- bootEst(CAboot, MFboot) ###Bootstrap estimate for both CAboot and MFboot

BSCA.MF<-colMeans(CA.MFboot) ###Mean of Bootstrap value

BSCA.MF

CA.CAM<-CA.MFboot[,2] #Extract the require column of the matrix

bootCI(rCA.MF[2],CA.CAM)

###Activity Overlap######

### Overlap between Golden jackal (CA) and Caucasian squirrel (SA)

min(length(CA), length(SA)) #Checking minimum length from both species

rCA.SA <- overlapEst(CA,SA) ###Overlap for both species by using overlapEst function

rCA.SA

#b1 (Dhat1) for lower than 75 observation

#b4 (Dhat4) for higher than 75 observation

#####Drawing overlap plot CA vs SA

overlapPlot(CA,SA, main="",ylim=c(0,0.15),cex.axis=1.5,cex.lab=1.5,cex.main=1.5, xlab = "", ylab = "")

legend('topleft', c("Golden jackal", "Caucasian squirrel"), lty=c(1,2), col=c(1,4), bty='n',cex=1.5)

text(x = 21, y = 0.145, labels = sprintf("Dhat1: 0.28"), col = "black", cex = 1.3)

par(mar = c(3, 3, 2, 1))

###To estimate confidence intervals we need to know the sampling distribution

###which our coefficient of overlapping is drawn from,

###i.e, the distribution we would get if we had a very large number of independent samples from nature.

###The best way to investigate this is to use a bootstrap.

CAboot<-resample(CA,1000) ###2000 times Bootstrap for Golden jackal (CA) by using resample function

SAboot<-resample(SA,1000) ###2000 times Bootstrap for Caucasian squirrel (SA) by using resample function

CA.SAboot <- bootEst(CAboot, SAboot) ###Bootstrap estimate for both CAboot and SAboot

BSCA.SA<-colMeans(CA.SAboot) ###Mean of Bootstrap value

BSCA.SA

CA.CAM<-CA.SAboot[,2] #Extract the require column of the matrix

bootCI(rCA.SA[2],CA.CAM)

###Activity Overlap######

### Overlap between Golden jackal (CA) and Wildcat (FS)

min(length(CA), length(FS)) #Checking minimum length from both species

rCA.FS <- overlapEst(CA,FS) ###Overlap for both species by using overlapEst function

rCA.FS

#b1 (Dhat1) for lower than 75 observation

#b4 (Dhat4) for higher than 75 observation

#####Drawing overlap plot CA vs FS

overlapPlot(CA,FS, main="",ylim=c(0,0.15),cex.axis=1.5,cex.lab=1.5,cex.main=1.5, xlab = "", ylab = "")

legend('topleft', c("Golden jackal", "Wildcat"), lty=c(1,2), col=c(1,4), bty='n',cex=1.5)

text(x = 21, y = 0.145, labels = sprintf("Dhat1: 0.61"), col = "black", cex = 1.3)

par(mar = c(3, 3, 2, 1))

###To estimate confidence intervals we need to know the sampling distribution

###which our coefficient of overlapping is drawn from,

###i.e, the distribution we would get if we had a very large number of independent samples from nature.

###The best way to investigate this is to use a bootstrap.

CAboot<-resample(CA,1000) ###2000 times Bootstrap for Golden jackal (CA) by using resample function

FSboot<-resample(FS,1000) ###2000 times Bootstrap for Wildcat (FS) by using resample function

CA.FSboot <- bootEst(CAboot, FSboot) ###Bootstrap estimate for both CAboot and FSboot

BSCA.FS<-colMeans(CA.FSboot) ###Mean of Bootstrap value

BSCA.FS

CA.CAM<-CA.FSboot[,2] #Extract the require column of the matrix

bootCI(rCA.FS[2],CA.CAM)

###Activity Overlap######

### Overlap between Golden jackal (CA) and European hare (LE)

min(length(CA), length(LE)) #Checking minimum length from both species

rCA.LE <- overlapEst(CA,LE) ###Overlap for both species by using overlapEst function

rCA.LE

#b1 (Dhat1) for lower than 75 observation

#b4 (Dhat4) for higher than 75 observation

#####Drawing overlap plot CA vs LE

overlapPlot(CA,LE, main="",ylim=c(0,0.15),cex.axis=1.5,cex.lab=1.5,cex.main=1.5, xlab = "", ylab = "")

legend('topleft', c("Golden jackal", "European hare"), lty=c(1,2), col=c(1,4), bty='n',cex=1.5)

text(x = 21, y = 0.145, labels = sprintf("Dhat1: 0.69"), col = "black", cex = 1.3)

par(mar = c(3, 3, 2, 1))

###To estimate confidence intervals we need to know the sampling distribution

###which our coefficient of overlapping is drawn from,

###i.e, the distribution we would get if we had a very large number of independent samples from nature.

###The best way to investigate this is to use a bootstrap.

CAboot<-resample(CA,1000) ###2000 times Bootstrap for Golden jackal (CA) by using resample function

LEboot<-resample(LE,1000) ###2000 times Bootstrap for European hare (LE) by using resample function

CA.LEboot <- bootEst(CAboot, LEboot) ###Bootstrap estimate for both CAboot and LEboot

BSCA.LE<-colMeans(CA.LEboot) ###Mean of Bootstrap value

BSCA.LE

CA.CAM<-CA.LEboot[,2] #Extract the require column of the matrix

bootCI(rCA.LE[2],CA.CAM)

###Activity Overlap######

### Overlap between Golden jackal (CA) and European badger (MM)

min(length(CA), length(MM)) #Checking minimum length from both species

rCA.MM <- overlapEst(CA,MM) ###Overlap for both species by using overlapEst function

rCA.MM

#b1 (Dhat1) for lower than 75 observation

#b4 (Dhat4) for higher than 75 observation

#####Drawing overlap plot CA vs MM

overlapPlot(CA,MM, main="",ylim=c(0,0.15),cex.axis=1.5,cex.lab=1.5,cex.main=1.5, xlab = "", ylab = "")

legend('topleft', c("Golden jackal", "European badger"), lty=c(1,2), col=c(1,4), bty='n',cex=1.5)

text(x = 21, y = 0.145, labels = sprintf("Dhat1: 0.63"), col = "black", cex = 1.3)

par(mar = c(3, 3, 2, 1))

###To estimate confidence intervals we need to know the sampling distribution

###which our coefficient of overlapping is drawn from,

###i.e, the distribution we would get if we had a very large number of independent samples from nature.

###The best way to investigate this is to use a bootstrap.

CAboot<-resample(CA,1000) ###2000 times Bootstrap for Golden jackal (CA) by using resample function

MMboot<-resample(MM,1000) ###2000 times Bootstrap for European badger (MM) by using resample function

CA.MMboot <- bootEst(CAboot, MMboot) ###Bootstrap estimate for both CAboot and MMboot

BSCA.MM<-colMeans(CA.MMboot) ###Mean of Bootstrap value

BSCA.MM

CA.CAM<-CA.MMboot[,2] #Extract the require column of the matrix

bootCI(rCA.MM[2],CA.CAM)

###Activity Overlap######

### Overlap between Golden jackal (CA) and Brown bear (UA)

min(length(CA), length(UA)) #Checking minimum length from both species

rCA.UA <- overlapEst(CA,UA) ###Overlap for both species by using overlapEst function

rCA.UA

#b1 (Dhat1) for lower than 75 observation

#b4 (Dhat4) for higher than 75 observation

#####Drawing overlap plot CA vs UA

overlapPlot(CA,UA, main="Golden jackal Vs Brown bear",ylim=c(0,0.20),cex.axis=1.5,cex.lab=1.5,cex.main=1.5, xlab = "", ylab = "")

legend('topleft', c("Golden jackal", "Brown bear"), lty=c(1,2), col=c(1,4), bty='n',cex=1.5)

text(x = 22, y = 0.08, labels = sprintf("Dhat1: 0.77"), col = "black", cex = 1.2)

par(mar = c(3, 3, 2, 2))

###To estimate confidence intervals we need to know the sampling distribution

###which our coefficient of overlapping is drawn from,

###i.e, the distribution we would get if we had a very large number of independent samples from nature.

###The best way to investigate this is to use a bootstrap.

CAboot<-resample(CA,1000) ###2000 times Bootstrap for Golden jackal (CA) by using resample function

UAboot<-resample(UA,1000) ###2000 times Bootstrap for Brown bear (UA) by using resample function

CA.UAboot <- bootEst(CAboot, UAboot) ###Bootstrap estimate for both CAboot and UAboot

BSCA.UA<-colMeans(CA.UAboot) ###Mean of Bootstrap value

BSCA.UA

CA.CAM<-CA.UAboot[,2] #Extract the require column of the matrix

bootCI(rCA.UA[2],CA.CAM)

###Activity Overlap######

### Overlap between Golden jackal (CA) and Wild boar (SS)

min(length(CA), length(SS)) #Checking minimum length from both species

rCA.SS <- overlapEst(CA,SS) ###Overlap for both species by using overlapEst function

rCA.SS

#b1 (Dhat1) for lower than 75 observation

#b4 (Dhat4) for higher than 75 observation

#####Drawing overlap plot CA vs SS

overlapPlot(CA,SS, main="",ylim=c(0,0.20),cex.axis=1.5,cex.lab=1.5,cex.main=1.5, xlab = "", ylab = "")

legend('topleft', c("Golden jackal", "Wild boar"), lty=c(1,2), col=c(1,4), bty='n',cex=1.5)

text(x = 21, y = 0.19, labels = sprintf("Dhat1: 0.66"), col = "black", cex = 1.3)

par(mar = c(3, 3, 2, 1))

###To estimate confidence intervals we need to know the sampling distribution

###which our coefficient of overlapping is drawn from,

###i.e, the distribution we would get if we had a very large number of independent samples from nature.

###The best way to investigate this is to use a bootstrap.

CAboot<-resample(CA,1000) ###2000 times Bootstrap for Golden jackal (CA) by using resample function

SSboot<-resample(SS,1000) ###2000 times Bootstrap for Wild boar (SS) by using resample function

CA.SSboot <- bootEst(CAboot, SSboot) ###Bootstrap estimate for both CAboot and SSboot

BSCA.SS<-colMeans(CA.SSboot) ###Mean of Bootstrap value

BSCA.SS

CA.CAM<-CA.SSboot[,2] #Extract the require column of the matrix

bootCI(rCA.SS[2],CA.CAM)

###Activity Overlap######

### Overlap between Roe deer (CC) and Southern white-breasted hedgehog (EC)

min(length(CC), length(EC)) #Checking minimum length from both species

rCC.EC <- overlapEst(CC,EC) ###Overlap for both species by using overlapEst function

rCC.EC

#b1 (Dhat1) for lower than 75 observation

#b4 (Dhat4) for higher than 75 observation

#####Drawing overlap plot CC vs EC

overlapPlot(CC,EC, main="",ylim=c(0,0.15),cex.axis=1.5,cex.lab=1.5,cex.main=1.5, xlab = "", ylab = "")

legend('topleft', c("Roe deer", "Hedgehog"), lty=c(1,2), col=c(1,4), bty='n',cex=1.5)

text(x = 21, y = 0.145, labels = sprintf("Dhat1: 0.39"), col = "black", cex = 1.3)

par(mar = c(3, 3, 2, 1))

###To estimate confidence intervals we need to know the sampling distribution

###which our coefficient of overlapping is drawn from,

###i.e, the distribution we would get if we had a very large number of independent samples from nature.

###The best way to investigate this is to use a bootstrap.

CCboot<-resample(CC,1000) ###2000 times Bootstrap for Roe deer (CC) by using resample function

ECboot<-resample(EC,1000) ###2000 times Bootstrap for Southern white-breasted hedgehog (EC) by using resample function

CC.ECboot <- bootEst(CCboot, ECboot) ###Bootstrap estimate for both CCboot and ECboot

BSCC.EC<-colMeans(CC.ECboot) ###Mean of Bootstrap value

BSCC.EC

CC.CAM<-CC.ECboot[,2] #Extract the require column of the matrix

bootCI(rCC.EC[2],CC.CAM)

###Activity Overlap######

### Overlap between Roe deer (CC) and Caucasian squirrel (SA)

min(length(CC), length(SA)) #Checking minimum length from both species

rCC.SA <- overlapEst(CC,SA) ###Overlap for both species by using overlapEst function

rCC.SA

#b1 (Dhat1) for lower than 75 observation

#b4 (Dhat4) for higher than 75 observation

#####Drawing overlap plot CC vs SA

overlapPlot(CC,SA, main="",ylim=c(0,0.15),cex.axis=1.5,cex.lab=1.5,cex.main=1.5, xlab = "", ylab = "")

legend('topleft', c("Roe deer", "Caucasian squirrel"), lty=c(1,2), col=c(1,4), bty='n',cex=1.5)

text(x = 21, y = 0.145, labels = sprintf("Dhat1: 0.57"), col = "black", cex = 1.3)

par(mar = c(3, 3, 2, 1))

###To estimate confidence intervals we need to know the sampling distribution

###which our coefficient of overlapping is drawn from,

###i.e, the distribution we would get if we had a very large number of independent samples from nature.

###The best way to investigate this is to use a bootstrap.

CCboot<-resample(CC,1000) ###2000 times Bootstrap for Roe deer (CC) by using resample function

SAboot<-resample(SA,1000) ###2000 times Bootstrap for Caucasian squirrel (SA) by using resample function

CC.SAboot <- bootEst(CCboot, SAboot) ###Bootstrap estimate for both CCboot and SAboot

BSCC.SA<-colMeans(CC.SAboot) ###Mean of Bootstrap value

BSCC.SA

CC.CAM<-CC.SAboot[,2] #Extract the require column of the matrix

bootCI(rCC.SA[2],CC.CAM)

###Activity Overlap######

### Overlap between Roe deer (CC) and Wildcat (FS)

min(length(CC), length(FS)) #Checking minimum length from both species

rCC.FS <- overlapEst(CC,FS) ###Overlap for both species by using overlapEst function

rCC.FS

#b1 (Dhat1) for lower than 75 observation

#b4 (Dhat4) for higher than 75 observation

#####Drawing overlap plot CC vs FS

overlapPlot(CC,FS, main="",ylim=c(0,0.15),cex.axis=1.5,cex.lab=1.5,cex.main=1.5, xlab = "", ylab = "")

legend('topleft', c("Roe deer", "Wildcat"), lty=c(1,2), col=c(1,4), bty='n',cex=1.5)

text(x = 21, y = 0.145, labels = sprintf("Dhat1: 0.55"), col = "black", cex = 1.3)

par(mar = c(3, 3, 2, 1))

###To estimate confidence intervals we need to know the sampling distribution

###which our coefficient of overlapping is drawn from,

###i.e, the distribution we would get if we had a very large number of independent samples from nature.

###The best way to investigate this is to use a bootstrap.

CCboot<-resample(CC,1000) ###2000 times Bootstrap for Roe deer (CC) by using resample function

FSboot<-resample(FS,1000) ###2000 times Bootstrap for Wildcat (FS) by using resample function

CC.FSboot <- bootEst(CCboot, FSboot) ###Bootstrap estimate for both CCboot and FSboot

BSCC.FS<-colMeans(CC.FSboot) ###Mean of Bootstrap value

BSCC.FS

CC.CAM<-CC.FSboot[,2] #Extract the require column of the matrix

bootCI(rCC.FS[2],CC.CAM)

###Activity Overlap######

### Overlap between Roe deer (CC) and European hare (LE)

min(length(CC), length(LE)) #Checking minimum length from both species

rCC.LE <- overlapEst(CC,LE) ###Overlap for both species by using overlapEst function

rCC.LE

#b1 (Dhat1) for lower than 75 observation

#b4 (Dhat4) for higher than 75 observation

#####Drawing overlap plot CC vs LE

overlapPlot(CC,LE, main="",ylim=c(0,0.15),cex.axis=1.5,cex.lab=1.5,cex.main=1.5, xlab = "", ylab = "")

legend('topleft', c("Roe deer", "European hare"), lty=c(1,2), col=c(1,4), bty='n',cex=1.5)

text(x = 21, y = 0.145, labels = sprintf("Dhat1: 0.46"), col = "black", cex = 1.3)

par(mar = c(3, 3, 2, 1))

###To estimate confidence intervals we need to know the sampling distribution

###which our coefficient of overlapping is drawn from,

###i.e, the distribution we would get if we had a very large number of independent samples from nature.

###The best way to investigate this is to use a bootstrap.

CCboot<-resample(CC,1000) ###2000 times Bootstrap for Roe deer (CC) by using resample function

LEboot<-resample(LE,1000) ###2000 times Bootstrap for European hare (LE) by using resample function

CC.LEboot <- bootEst(CCboot, LEboot) ###Bootstrap estimate for both CCboot and LEboot

BSCC.LE<-colMeans(CC.LEboot) ###Mean of Bootstrap value

BSCC.LE

CC.CAM<-CC.LEboot[,2] #Extract the require column of the matrix

bootCI(rCC.LE[2],CC.CAM)

###Activity Overlap######

### Overlap between Roe deer (CC) and European badger (MM)

min(length(CC), length(MM)) #Checking minimum length from both species

rCC.MM <- overlapEst(CC,MM) ###Overlap for both species by using overlapEst function

rCC.MM

#b1 (Dhat1) for lower than 75 observation

#b4 (Dhat4) for higher than 75 observation

#####Drawing overlap plot CC vs MM

overlapPlot(CC,MM, main="",ylim=c(0,0.15),cex.axis=1.5,cex.lab=1.5,cex.main=1.5, xlab = "", ylab = "")

legend('topleft', c("Roe deer", "European badger"), lty=c(1,2), col=c(1,4), bty='n',cex=1.5)

text(x = 21, y = 0.145, labels = sprintf("Dhat1: 0.41"), col = "black", cex = 1.3)

par(mar = c(3, 3, 2, 1))

###To estimate confidence intervals we need to know the sampling distribution

###which our coefficient of overlapping is drawn from,

###i.e, the distribution we would get if we had a very large number of independent samples from nature.

###The best way to investigate this is to use a bootstrap.

CCboot<-resample(CC,1000) ###2000 times Bootstrap for Roe deer (CC) by using resample function

MMboot<-resample(MM,1000) ###2000 times Bootstrap for European badger (MM) by using resample function

CC.MMboot <- bootEst(CCboot, MMboot) ###Bootstrap estimate for both CCboot and MMboot

BSCC.MM<-colMeans(CC.MMboot) ###Mean of Bootstrap value

BSCC.MM

CC.CAM<-CC.MMboot[,2] #Extract the require column of the matrix

bootCI(rCC.MM[2],CC.CAM)

###Activity Overlap######

### Overlap between Roe deer (CC) and Wild boar (SS)

min(length(CC), length(SS)) #Checking minimum length from both species

rCC.SS <- overlapEst(CC,SS) ###Overlap for both species by using overlapEst function

rCC.SS

#b1 (Dhat1) for lower than 75 observation

#b4 (Dhat4) for higher than 75 observation

#####Drawing overlap plot CC vs SS

overlapPlot(CC,SS, main="",ylim=c(0,0.15),cex.axis=1.5,cex.lab=1.5,cex.main=1.5, xlab = "", ylab = "")

legend('topleft', c("Roe deer", "Wild boar"), lty=c(1,2), col=c(1,4), bty='n',cex=1.5)

text(x = 21, y = 0.145, labels = sprintf("Dhat1: 0.50"), col = "black", cex = 1.3)

par(mar = c(3, 3, 2, 1))

###To estimate confidence intervals we need to know the sampling distribution

###which our coefficient of overlapping is drawn from,

###i.e, the distribution we would get if we had a very large number of independent samples from nature.

###The best way to investigate this is to use a bootstrap.

CCboot<-resample(CC,1000) ###2000 times Bootstrap for Roe deer (CC) by using resample function

SSboot<-resample(SS,1000) ###2000 times Bootstrap for Wild boar (SS) by using resample function

CC.SSboot <- bootEst(CCboot, SSboot) ###Bootstrap estimate for both CCboot and SSboot

BSCC.SS<-colMeans(CC.SSboot) ###Mean of Bootstrap value

BSCC.SS

CC.CAM<-CC.SSboot[,2] #Extract the require column of the matrix

bootCI(rCC.SS[2],CC.CAM)

###Activity Overlap######

### Overlap between Southern white-breasted hedgehog (EC) and Beech marten (MF)

min(length(EC), length(MF)) #Checking minimum length from both species

rEC.MF <- overlapEst(EC,MF) ###Overlap for both species by using overlapEst function

rEC.MF

#b1 (Dhat1) for lower than 75 observation

#b4 (Dhat4) for higher than 75 observation

#####Drawing overlap plot EC vs MF

overlapPlot(EC,MF, main="",ylim=c(0,0.15),cex.axis=1.5,cex.lab=1.5,cex.main=1.5, xlab = "", ylab = "")

legend('topleft', c("Hedgehog", "Beech marten"), lty=c(1,2), col=c(1,4), bty='n',cex=1.5)

text(x = 21, y = 0.145, labels = sprintf("Dhat4: 0.84"), col = "black", cex = 1.3)

par(mar = c(3, 3, 2, 1))

###To estimate confidence intervals we need to know the sampling distribution

###which our coefficient of overlapping is drawn from,

###i.e, the distribution we would get if we had a very large number of independent samples from nature.

###The best way to investigate this is to use a bootstrap.

ECboot<-resample(EC,1000) ###2000 times Bootstrap for Southern white-breasted hedgehog (EC) by using resample function

MFboot<-resample(MF,1000) ###2000 times Bootstrap for Beech marten (MF) by using resample function

EC.MFboot <- bootEst(ECboot, MFboot) ###Bootstrap estimate for both ECboot and MFboot

BSEC.MF<-colMeans(EC.MFboot) ###Mean of Bootstrap value

BSEC.MF

EC.CAM<-EC.MFboot[,2] #Extract the require column of the matrix

bootCI(rEC.MF[2],EC.CAM)

###Activity Overlap######

### Overlap between Southern white-breasted hedgehog (EC) and Caucasian squirrel (SA)

min(length(EC), length(SA)) #Checking minimum length from both species

rEC.SA <- overlapEst(EC,SA) ###Overlap for both species by using overlapEst function

rEC.SA

#b1 (Dhat1) for lower than 75 observation

#b4 (Dhat4) for higher than 75 observation

#####Drawing overlap plot EC vs SA

overlapPlot(EC,SA, main="",ylim=c(0,0.15),cex.axis=1.5,cex.lab=1.5,cex.main=1.5, xlab = "", ylab = "")

legend('topleft', c("Hedgehog", "Caucasian squirrel"), lty=c(1,2), col=c(1,4), bty='n',cex=1.5)

text(x = 21, y = 0.145, labels = sprintf("Dhat1: 0.081"), col = "black", cex = 1.3)

par(mar = c(3, 3, 2, 1))

###To estimate confidence intervals we need to know the sampling distribution

###which our coefficient of overlapping is drawn from,

###i.e, the distribution we would get if we had a very large number of independent samples from nature.

###The best way to investigate this is to use a bootstrap.

ECboot<-resample(EC,1000) ###2000 times Bootstrap for Southern white-breasted hedgehog (EC) by using resample function

SAboot<-resample(SA,1000) ###2000 times Bootstrap for Caucasian squirrel (SA) by using resample function

EC.SAboot <- bootEst(ECboot, SAboot) ###Bootstrap estimate for both ECboot and SAboot

BSEC.SA<-colMeans(EC.SAboot) ###Mean of Bootstrap value

BSEC.SA

EC.CAM<-EC.SAboot[,2] #Extract the require column of the matrix

bootCI(rEC.SA[2],EC.CAM)

###Activity Overlap######

### Overlap between Southern white-breasted hedgehog (EC) and Wildcat (FS)

min(length(EC), length(FS)) #Checking minimum length from both species

rEC.FS <- overlapEst(EC,FS) ###Overlap for both species by using overlapEst function

rEC.FS

#b1 (Dhat1) for lower than 75 observation

#b4 (Dhat4) for higher than 75 observation

#####Drawing overlap plot EC vs FS

overlapPlot(EC,FS, main="",ylim=c(0,0.15),cex.axis=1.5,cex.lab=1.5,cex.main=1.5, xlab = "", ylab = "")

legend('topleft', c("Hedgehog", "Wildcat"), lty=c(1,2), col=c(1,4), bty='n',cex=1.5)

text(x = 21, y = 0.145, labels = sprintf("Dhat1: 0.64"), col = "black", cex = 1.3)

par(mar = c(3, 3, 2, 1))

###To estimate confidence intervals we need to know the sampling distribution

###which our coefficient of overlapping is drawn from,

###i.e, the distribution we would get if we had a very large number of independent samples from nature.

###The best way to investigate this is to use a bootstrap.

ECboot<-resample(EC,1000) ###2000 times Bootstrap for Southern white-breasted hedgehog (EC) by using resample function

FSboot<-resample(FS,1000) ###2000 times Bootstrap for Wildcat (FS) by using resample function

EC.FSboot <- bootEst(ECboot, FSboot) ###Bootstrap estimate for both ECboot and FSboot

BSEC.FS<-colMeans(EC.FSboot) ###Mean of Bootstrap value

BSEC.FS

EC.CAM<-EC.FSboot[,2] #Extract the require column of the matrix

bootCI(rEC.FS[2],EC.CAM)

###Activity Overlap######

### Overlap between Southern white-breasted hedgehog (EC) and European hare (LE)

min(length(EC), length(LE)) #Checking minimum length from both species

rEC.LE <- overlapEst(EC,LE) ###Overlap for both species by using overlapEst function

rEC.LE

#b1 (Dhat1) for lower than 75 observation

#b4 (Dhat4) for higher than 75 observation

#####Drawing overlap plot EC vs LE

overlapPlot(EC,LE, main="",ylim=c(0,0.15),cex.axis=1.5,cex.lab=1.5,cex.main=1.5, xlab = "", ylab = "")

legend('topleft', c("Hedgehog", "European hare"), lty=c(1,2), col=c(1,4), bty='n',cex=1.5)

text(x = 21, y = 0.145, labels = sprintf("Dhat1: 0.74"), col = "black", cex = 1.3)

par(mar = c(3, 3, 2, 1))

###To estimate confidence intervals we need to know the sampling distribution

###which our coefficient of overlapping is drawn from,

###i.e, the distribution we would get if we had a very large number of independent samples from nature.

###The best way to investigate this is to use a bootstrap.

ECboot<-resample(EC,1000) ###2000 times Bootstrap for Southern white-breasted hedgehog (EC) by using resample function

LEboot<-resample(LE,1000) ###2000 times Bootstrap for European hare (LE) by using resample function

EC.LEboot <- bootEst(ECboot, LEboot) ###Bootstrap estimate for both ECboot and LEboot

BSEC.LE<-colMeans(EC.LEboot) ###Mean of Bootstrap value

BSEC.LE

EC.CAM<-EC.LEboot[,2] #Extract the require column of the matrix

bootCI(rEC.LE[2],EC.CAM)

###Activity Overlap######

### Overlap between Southern white-breasted hedgehog (EC) and European badger (MM)

min(length(EC), length(MM)) #Checking minimum length from both species

rEC.MM <- overlapEst(EC,MM) ###Overlap for both species by using overlapEst function

rEC.MM

#b1 (Dhat1) for lower than 75 observation

#b4 (Dhat4) for higher than 75 observation

#####Drawing overlap plot EC vs MM

overlapPlot(EC,MM, main="",ylim=c(0,0.15),cex.axis=1.5,cex.lab=1.5,cex.main=1.5, xlab = "", ylab = "")

legend('topleft', c("Hedgehog", "European badger"), lty=c(1,2), col=c(1,4), bty='n',cex=1.5)

text(x = 21, y = 0.145, labels = sprintf("Dhat1: 0.75"), col = "black", cex = 1.3)

par(mar = c(3, 3, 2, 1))

###To estimate confidence intervals we need to know the sampling distribution

###which our coefficient of overlapping is drawn from,

###i.e, the distribution we would get if we had a very large number of independent samples from nature.

###The best way to investigate this is to use a bootstrap.

ECboot<-resample(EC,1000) ###2000 times Bootstrap for Southern white-breasted hedgehog (EC) by using resample function

MMboot<-resample(MM,1000) ###2000 times Bootstrap for European badger (MM) by using resample function

EC.MMboot <- bootEst(ECboot, MMboot) ###Bootstrap estimate for both ECboot and MMboot

BSEC.MM<-colMeans(EC.MMboot) ###Mean of Bootstrap value

BSEC.MM

EC.CAM<-EC.MMboot[,2] #Extract the require column of the matrix

bootCI(rEC.MM[2],EC.CAM)

###Activity Overlap######

### Overlap between Southern white-breasted hedgehog (EC) and Wild boar (SS)

min(length(EC), length(SS)) #Checking minimum length from both species

rEC.SS <- overlapEst(EC,SS) ###Overlap for both species by using overlapEst function

rEC.SS

#b1 (Dhat1) for lower than 75 observation

#b4 (Dhat4) for higher than 75 observation

#####Drawing overlap plot EC vs SS

overlapPlot(EC,SS, main="",ylim=c(0,0.15),cex.axis=1.5,cex.lab=1.5,cex.main=1.5, xlab = "", ylab = "")

legend('topleft', c("Hedgehog", "Wild boar"), lty=c(1,2), col=c(1,4), bty='n',cex=1.5)

text(x = 21, y = 0.145, labels = sprintf("Dhat1: 0.68"), col = "black", cex = 1.3)

par(mar = c(3, 3, 2, 1))

###To estimate confidence intervals we need to know the sampling distribution

###which our coefficient of overlapping is drawn from,

###i.e, the distribution we would get if we had a very large number of independent samples from nature.

###The best way to investigate this is to use a bootstrap.

ECboot<-resample(EC,1000) ###2000 times Bootstrap for Southern white-breasted hedgehog (EC) by using resample function

SSboot<-resample(SS,1000) ###2000 times Bootstrap for Wild boar (SS) by using resample function

EC.SSboot <- bootEst(ECboot, SSboot) ###Bootstrap estimate for both ECboot and SSboot

BSEC.SS<-colMeans(EC.SSboot) ###Mean of Bootstrap value

BSEC.SS

EC.CAM<-EC.SSboot[,2] #Extract the require column of the matrix

bootCI(rEC.SS[2],EC.CAM)

###Activity Overlap######

### Overlap between Beech marten (MF) and Caucasian squirrel (SA)

min(length(MF), length(SA)) #Checking minimum length from both species

rMF.SA <- overlapEst(MF,SA) ###Overlap for both species by using overlapEst function

rMF.SA

#b1 (Dhat1) for lower than 75 observation

#b4 (Dhat4) for higher than 75 observation

#####Drawing overlap plot MF vs SA

overlapPlot(MF,SA, main="",ylim=c(0,0.15),cex.axis=1.5,cex.lab=1.5,cex.main=1.5, xlab = "", ylab = "")

legend('topleft', c("Beech marten", "Caucasian squirrel"), lty=c(1,2), col=c(1,4), bty='n',cex=1.5)

text(x = 21, y = 0.145, labels = sprintf("Dhat1: 0.15"), col = "black", cex = 1.3)

par(mar = c(3, 3, 2, 1))

###To estimate confidence intervals we need to know the sampling distribution

###which our coefficient of overlapping is drawn from,

###i.e, the distribution we would get if we had a very large number of independent samples from nature.

###The best way to investigate this is to use a bootstrap.

MFboot<-resample(MF,1000) ###2000 times Bootstrap for Beech marten (MF) by using resample function

SAboot<-resample(SA,1000) ###2000 times Bootstrap for Caucasian squirrel (SA) by using resample function

MF.SAboot <- bootEst(MFboot, SAboot) ###Bootstrap estimate for both MFboot and SAboot

BSMF.SA<-colMeans(MF.SAboot) ###Mean of Bootstrap value

BSMF.SA

MF.CAM<-MF.SAboot[,2] #Extract the require column of the matrix

bootCI(rMF.SA[2],MF.CAM)

###Activity Overlap######

### Overlap between Beech marten (MF) and European hare (LE)

min(length(MF), length(LE)) #Checking minimum length from both species

rMF.LE <- overlapEst(MF,LE) ###Overlap for both species by using overlapEst function

rMF.LE

#b1 (Dhat1) for lower than 75 observation

#b4 (Dhat4) for higher than 75 observation

#####Drawing overlap plot MF vs LE

overlapPlot(MF,LE, main="",ylim=c(0,0.15),cex.axis=1.5,cex.lab=1.5,cex.main=1.5, xlab = "", ylab = "")

legend('topleft', c("Beech marten", "European hare"), lty=c(1,2), col=c(1,4), bty='n',cex=1.5)

text(x = 21, y = 0.145, labels = sprintf("Dhat1: 0.73"), col = "black", cex = 1.3)

par(mar = c(3, 3, 2, 1))

###To estimate confidence intervals we need to know the sampling distribution

###which our coefficient of overlapping is drawn from,

###i.e, the distribution we would get if we had a very large number of independent samples from nature.

###The best way to investigate this is to use a bootstrap.

MFboot<-resample(MF,1000) ###2000 times Bootstrap for Beech marten (MF) by using resample function

LEboot<-resample(LE,1000) ###2000 times Bootstrap for European hare (LE) by using resample function

MF.LEboot <- bootEst(MFboot, LEboot) ###Bootstrap estimate for both MFboot and LEboot

BSMF.LE<-colMeans(MF.LEboot) ###Mean of Bootstrap value

BSMF.LE

MF.CAM<-MF.LEboot[,2] #Extract the require column of the matrix

bootCI(rMF.LE[2],MF.CAM)

###Activity Overlap######

### Overlap between Beech marten (MF) and European badger (MM)

min(length(MF), length(MM)) #Checking minimum length from both species

rMF.MM <- overlapEst(MF,MM) ###Overlap for both species by using overlapEst function

rMF.MM

#b1 (Dhat1) for lower than 75 observation

#b4 (Dhat4) for higher than 75 observation

#####Drawing overlap plot MF vs MM

overlapPlot(MF,MM, main="",ylim=c(0,0.15),cex.axis=1.5,cex.lab=1.5,cex.main=1.5, xlab = "", ylab = "")

legend('topleft', c("Beech marten", "European badger"), lty=c(1,2), col=c(1,4), bty='n',cex=1.5)

text(x = 21, y = 0.145, labels = sprintf("Dhat1: 0.71"), col = "black", cex = 1.3)

par(mar = c(3, 3, 2, 1))

###To estimate confidence intervals we need to know the sampling distribution

###which our coefficient of overlapping is drawn from,

###i.e, the distribution we would get if we had a very large number of independent samples from nature.

###The best way to investigate this is to use a bootstrap.

MFboot<-resample(MF,1000) ###2000 times Bootstrap for Beech marten (MF) by using resample function

MMboot<-resample(MM,1000) ###2000 times Bootstrap for European badger (MM) by using resample function

MF.MMboot <- bootEst(MFboot, MMboot) ###Bootstrap estimate for both MFboot and MMboot

BSMF.MM<-colMeans(MF.MMboot) ###Mean of Bootstrap value

BSMF.MM

MF.CAM<-MF.MMboot[,2] #Extract the require column of the matrix

bootCI(rMF.MM[2],MF.CAM)

###Activity Overlap######

### Overlap between Beech marten (MF) and Roe deer (cc)

min(length(MF), length(CC)) #Checking minimum length from both species

rMF.CC <- overlapEst(MF,CC) ###Overlap for both species by using overlapEst function

rMF.CC

#b1 (Dhat1) for lower than 75 observation

#b4 (Dhat4) for higher than 75 observation

#####Drawing overlap plot MF vs CC

overlapPlot(MF,CC, main="",ylim=c(0,0.15),cex.axis=1.5,cex.lab=1.5,cex.main=1.5, xlab = "", ylab = "")

legend('topleft', c("Beech marten", "Roe deer"), lty=c(1,2), col=c(1,4), bty='n',cex=1.5)

text(x = 21, y = 0.145, labels = sprintf("Dhat1: 0.48"), col = "black", cex = 1.3)

par(mar = c(3, 3, 2, 1))

###To estimate confidence intervals we need to know the sampling distribution

###which our coefficient of overlapping is drawn from,

###i.e, the distribution we would get if we had a very large number of independent samples from nature.

###The best way to investigate this is to use a bootstrap.

MFboot<-resample(MF,1000) ###2000 times Bootstrap for Beech marten (MF) by using resample function

CCboot<-resample(CC,1000) ###2000 times Bootstrap for Roe deer (CC) by using resample function

MF.CCboot <- bootEst(MFboot, CCboot) ###Bootstrap estimate for both MFboot and CCboot

BSMF.CC<-colMeans(MF.CCboot) ###Mean of Bootstrap value

BSMF.CC

MF.CAM<-MF.CCboot[,2] #Extract the require column of the matrix

bootCI(rMF.CC[2],MF.CAM)

###Activity Overlap######

### Overlap between Caucasian squirrel (SA) and Wildcat (FS)

min(length(SA), length(FS)) #Checking minimum length from both species

rSA.FS <- overlapEst(SA,FS) ###Overlap for both species by using overlapEst function

rSA.FS

#b1 (Dhat1) for lower than 75 observation

#b4 (Dhat4) for higher than 75 observation

#####Drawing overlap plot SA vs FS

overlapPlot(SA,FS, main="",ylim=c(0,0.15),cex.axis=1.5,cex.lab=1.5,cex.main=1.5, xlab = "", ylab = "")

legend('topleft', c("Caucasian squirrel", "Wildcat"), lty=c(1,2), col=c(1,4), bty='n',cex=1.5)

text(x = 21, y = 0.145, labels = sprintf("Dhat1: 0.29"), col = "black", cex = 1.3)

par(mar = c(3, 3, 2, 1))

###To estimate confidence intervals we need to know the sampling distribution

###which our coefficient of overlapping is drawn from,

###i.e, the distribution we would get if we had a very large number of independent samples from nature.

###The best way to investigate this is to use a bootstrap.

SAboot<-resample(SA,1000) ###2000 times Bootstrap for Caucasian squirrel (SA) by using resample function

FSboot<-resample(FS,1000) ###2000 times Bootstrap for Wildcat (FS) by using resample function

SA.FSboot <- bootEst(SAboot, FSboot) ###Bootstrap estimate for both SAboot and FSboot

BSSA.FS<-colMeans(SA.FSboot) ###Mean of Bootstrap value

BSSA.FS

SA.CAM<-SA.FSboot[,2] #Extract the require column of the matrix

bootCI(rSA.FS[2],SA.CAM)

###Activity Overlap######

### Overlap between Caucasian squirrel (SA) and European hare (LE)

min(length(SA), length(LE)) #Checking minimum length from both species

rSA.LE <- overlapEst(SA,LE) ###Overlap for both species by using overlapEst function

rSA.LE

#b1 (Dhat1) for lower than 75 observation

#b4 (Dhat4) for higher than 75 observation

#####Drawing overlap plot SA vs LE

overlapPlot(SA,LE, main="",ylim=c(0,0.15),cex.axis=1.5,cex.lab=1.5,cex.main=1.5, xlab = "", ylab = "")

legend('topleft', c("Caucasian squirrel", "European hare"), lty=c(1,2), col=c(1,4), bty='n',cex=1.5)

text(x = 21, y = 0.145, labels = sprintf("Dhat1: 0.14"), col = "black", cex = 1.3)

par(mar = c(3, 3, 2, 1))

###To estimate confidence intervals we need to know the sampling distribution

###which our coefficient of overlapping is drawn from,

###i.e, the distribution we would get if we had a very large number of independent samples from nature.

###The best way to investigate this is to use a bootstrap.

SAboot<-resample(SA,1000) ###2000 times Bootstrap for Caucasian squirrel (SA) by using resample function

LEboot<-resample(LE,1000) ###2000 times Bootstrap for European hare (LE) by using resample function

SA.LEboot <- bootEst(SAboot, LEboot) ###Bootstrap estimate for both SAboot and LEboot

BSSA.LE<-colMeans(SA.LEboot) ###Mean of Bootstrap value

BSSA.LE

SA.CAM<-SA.LEboot[,2] #Extract the require column of the matrix

bootCI(rSA.LE[2],SA.CAM)

###Activity Overlap######

### Overlap between Caucasian squirrel (SA) and European badger (MM)

min(length(SA), length(MM)) #Checking minimum length from both species

rSA.MM <- overlapEst(SA,MM) ###Overlap for both species by using overlapEst function

rSA.MM

#b1 (Dhat1) for lower than 75 observation

#b4 (Dhat4) for higher than 75 observation

#####Drawing overlap plot SA vs MM

overlapPlot(SA,MM, main="",ylim=c(0,0.15),cex.axis=1.5,cex.lab=1.5,cex.main=1.5, xlab = "", ylab = "")

legend('topleft', c("Caucasian squirrel", "European badger"), lty=c(1,2), col=c(1,4), bty='n',cex=1.5)

text(x = 21, y = 0.145, labels = sprintf("Dhat1: 0.13"), col = "black", cex = 1.3)

par(mar = c(3, 3, 2, 1))

###To estimate confidence intervals we need to know the sampling distribution

###which our coefficient of overlapping is drawn from,

###i.e, the distribution we would get if we had a very large number of independent samples from nature.

###The best way to investigate this is to use a bootstrap.

SAboot<-resample(SA,1000) ###2000 times Bootstrap for Caucasian squirrel (SA) by using resample function

MMboot<-resample(MM,1000) ###2000 times Bootstrap for European badger (MM) by using resample function

SA.MMboot <- bootEst(SAboot, MMboot) ###Bootstrap estimate for both SAboot and MMboot

BSSA.MM<-colMeans(SA.MMboot) ###Mean of Bootstrap value

BSSA.MM

SA.CAM<-SA.MMboot[,2] #Extract the require column of the matrix

bootCI(rSA.MM[2],SA.CAM)

###Activity Overlap######

### Overlap between Caucasian squirrel (SA) and Wild boar (SS)

min(length(SA), length(SS)) #Checking minimum length from both species

rSA.SS <- overlapEst(SA,SS) ###Overlap for both species by using overlapEst function

rSA.SS

#b1 (Dhat1) for lower than 75 observation

#b4 (Dhat4) for higher than 75 observation

#####Drawing overlap plot SA vs SS

overlapPlot(SA,SS, main="",ylim=c(0,0.15),cex.axis=1.5,cex.lab=1.5,cex.main=1.5, xlab = "", ylab = "")

legend('topleft', c("Caucasian squirrel", "Wild boar"), lty=c(1,2), col=c(1,4), bty='n',cex=1.5)

text(x = 21, y = 0.145, labels = sprintf("Dhat1: 0.15"), col = "black", cex = 1.3)

par(mar = c(3, 3, 2, 1))

###To estimate confidence intervals we need to know the sampling distribution

###which our coefficient of overlapping is drawn from,

###i.e, the distribution we would get if we had a very large number of independent samples from nature.

###The best way to investigate this is to use a bootstrap.

SAboot<-resample(SA,1000) ###2000 times Bootstrap for Caucasian squirrel (SA) by using resample function

SSboot<-resample(SS,1000) ###2000 times Bootstrap for Wild boar (SS) by using resample function

SA.SSboot <- bootEst(SAboot, SSboot) ###Bootstrap estimate for both SAboot and SSboot

BSSA.SS<-colMeans(SA.SSboot) ###Mean of Bootstrap value

BSSA.SS

SA.CAM<-SA.SSboot[,2] #Extract the require column of the matrix

bootCI(rSA.SS[2],SA.CAM)

###Activity Overlap######

### Overlap between Wildcat (FS) and European hare (LE)

min(length(FS), length(LE)) #Checking minimum length from both species

rFS.LE <- overlapEst(FS,LE) ###Overlap for both species by using overlapEst function

rFS.LE

#b1 (Dhat1) for lower than 75 observation

#b4 (Dhat4) for higher than 75 observation

#####Drawing overlap plot FS vs LE

overlapPlot(FS,LE, main="",ylim=c(0,0.15),cex.axis=1.5,cex.lab=1.5,cex.main=1.5, xlab = "", ylab = "")

legend('topleft', c("Wildcat", "European hare"), lty=c(1,2), col=c(1,4), bty='n',cex=1.5)

text(x = 21, y = 0.145, labels = sprintf("Dhat1: 0.59"), col = "black", cex = 1.3)

par(mar = c(3, 3, 2, 1))

###To estimate confidence intervals we need to know the sampling distribution

###which our coefficient of overlapping is drawn from,

###i.e, the distribution we would get if we had a very large number of independent samples from nature.

###The best way to investigate this is to use a bootstrap.

FSboot<-resample(FS,1000) ###2000 times Bootstrap for Wildcat (FS) by using resample function

LEboot<-resample(LE,1000) ###2000 times Bootstrap for European hare (LE) by using resample function

FS.LEboot <- bootEst(FSboot, LEboot) ###Bootstrap estimate for both FSboot and LEboot

BSFS.LE<-colMeans(FS.LEboot) ###Mean of Bootstrap value

BSFS.LE

FS.CAM<-FS.LEboot[,2] #Extract the require column of the matrix

bootCI(rFS.LE[2],FS.CAM)

###Activity Overlap######

### Overlap between Wildcat (FS) and European badger (MM)

min(length(FS), length(MM)) #Checking minimum length from both species

rFS.MM <- overlapEst(FS,MM) ###Overlap for both species by using overlapEst function

rFS.MM

#b1 (Dhat1) for lower than 75 observation

#b4 (Dhat4) for higher than 75 observation

#####Drawing overlap plot FS vs MM

overlapPlot(FS,MM, main="",ylim=c(0,0.15),cex.axis=1.5,cex.lab=1.5,cex.main=1.5, xlab = "", ylab = "")

legend('topleft', c("Wildcat", "European badger"), lty=c(1,2), col=c(1,4), bty='n',cex=1.5)

text(x = 21, y = 0.145, labels = sprintf("Dhat1: 0.56"), col = "black", cex = 1.3)

par(mar = c(3, 3, 2, 1))

###To estimate confidence intervals we need to know the sampling distribution

###which our coefficient of overlapping is drawn from,

###i.e, the distribution we would get if we had a very large number of independent samples from nature.

###The best way to investigate this is to use a bootstrap.

FSboot<-resample(FS,1000) ###2000 times Bootstrap for Wildcat (FS) by using resample function

MMboot<-resample(MM,1000) ###2000 times Bootstrap for European badger (MM) by using resample function

FS.MMboot <- bootEst(FSboot, MMboot) ###Bootstrap estimate for both FSboot and MMboot

BSFS.MM<-colMeans(FS.MMboot) ###Mean of Bootstrap value

BSFS.MM

FS.CAM<-FS.MMboot[,2] #Extract the require column of the matrix

bootCI(rFS.MM[2],FS.CAM)

###Activity Overlap######

### Overlap between Wildcat (FS) and Wild boar (SS)

min(length(FS), length(SS)) #Checking minimum length from both species

rFS.SS <- overlapEst(FS,SS) ###Overlap for both species by using overlapEst function

rFS.SS

#b1 (Dhat1) for lower than 75 observation

#b4 (Dhat4) for higher than 75 observation

#####Drawing overlap plot FS vs SS

overlapPlot(FS,SS, main="",ylim=c(0,0.15),cex.axis=1.5,cex.lab=1.5,cex.main=1.5, xlab = "", ylab = "")

legend('topleft', c("Wildcat", "Wild boar"), lty=c(1,2), col=c(1,4), bty='n',cex=1.5)

text(x = 21, y = 0.145, labels = sprintf("Dhat1: 0.64"), col = "black", cex = 1.3)

par(mar = c(3, 3, 2, 1))

###To estimate confidence intervals we need to know the sampling distribution

###which our coefficient of overlapping is drawn from,

###i.e, the distribution we would get if we had a very large number of independent samples from nature.

###The best way to investigate this is to use a bootstrap.

FSboot<-resample(FS,1000) ###2000 times Bootstrap for Wildcat (FS) by using resample function

SSboot<-resample(SS,1000) ###2000 times Bootstrap for Wild boar (SS) by using resample function

FS.SSboot <- bootEst(FSboot, SSboot) ###Bootstrap estimate for both FSboot and SSboot

BSFS.SS<-colMeans(FS.SSboot) ###Mean of Bootstrap value

BSFS.SS

FS.CAM<-FS.SSboot[,2] #Extract the require column of the matrix

bootCI(rFS.SS[2],FS.CAM)

###Activity Overlap######

### Overlap between European hare (LE) and European badger (MM)

min(length(LE), length(MM)) #Checking minimum length from both species

rLE.MM <- overlapEst(LE,MM) ###Overlap for both species by using overlapEst function

rLE.MM

#b1 (Dhat1) for lower than 75 observation

#b4 (Dhat4) for higher than 75 observation

#####Drawing overlap plot LE vs MM

overlapPlot(LE,MM, main="",ylim=c(0,0.15),cex.axis=1.5,cex.lab=1.5,cex.main=1.5, xlab = "", ylab = "")

legend('topleft', c("European hare", "European badger"), lty=c(1,2), col=c(1,4), bty='n',cex=1.5)

text(x = 21, y = 0.145, labels = sprintf("Dhat1: 0.67"), col = "black", cex = 1.3)

par(mar = c(3, 3, 2, 1))

###To estimate confidence intervals we need to know the sampling distribution

###which our coefficient of overlapping is drawn from,

###i.e, the distribution we would get if we had a very large number of independent samples from nature.

###The best way to investigate this is to use a bootstrap.

LEboot<-resample(LE,1000) ###2000 times Bootstrap for European hare (LE) by using resample function

MMboot<-resample(MM,1000) ###2000 times Bootstrap for European badger (MM) by using resample function

LE.MMboot <- bootEst(LEboot, MMboot) ###Bootstrap estimate for both LEboot and MMboot

BSLE.MM<-colMeans(LE.MMboot) ###Mean of Bootstrap value

BSLE.MM

LE.CAM<-LE.MMboot[,2] #Extract the require column of the matrix

bootCI(rLE.MM[2],LE.CAM)

###Activity Overlap######

### Overlap between European hare (LE) and Wild boar (SS)

min(length(LE), length(SS)) #Checking minimum length from both species

rLE.SS <- overlapEst(LE,SS) ###Overlap for both species by using overlapEst function

rLE.SS

#b1 (Dhat1) for lower than 75 observation

#b4 (Dhat4) for higher than 75 observation

#####Drawing overlap plot LE vs SS

overlapPlot(LE,SS, main="",ylim=c(0,0.15),cex.axis=1.5,cex.lab=1.5,cex.main=1.5, xlab = "", ylab = "")

legend('topleft', c("European hare", "Wild boar"), lty=c(1,2), col=c(1,4), bty='n',cex=1.5)

text(x = 21, y = 0.145, labels = sprintf("Dhat1: 0.68"), col = "black", cex = 1.3)

par(mar = c(3, 3, 2, 1))

###To estimate confidence intervals we need to know the sampling distribution

###which our coefficient of overlapping is drawn from,

###i.e, the distribution we would get if we had a very large number of independent samples from nature.

###The best way to investigate this is to use a bootstrap.

LEboot<-resample(LE,1000) ###2000 times Bootstrap for European hare (LE) by using resample function

SSboot<-resample(SS,1000) ###2000 times Bootstrap for Wild boar (SS) by using resample function

LE.SSboot <- bootEst(LEboot, SSboot) ###Bootstrap estimate for both LEboot and SSboot

BSLE.SS<-colMeans(LE.SSboot) ###Mean of Bootstrap value

BSLE.SS

LE.CAM<-LE.SSboot[,2] #Extract the require column of the matrix

bootCI(rLE.SS[2],LE.CAM)

###Activity Overlap######

### Overlap between European badger (MM) and Wild boar (SS)

min(length(MM), length(SS)) #Checking minimum length from both species

rMM.SS <- overlapEst(MM,SS) ###Overlap for both species by using overlapEst function

rMM.SS

#b1 (Dhat1) for lower than 75 observation

#b4 (Dhat4) for higher than 75 observation

#####Drawing overlap plot MM vs SS

overlapPlot(MM,SS, main="",ylim=c(0,0.15),cex.axis=1.5,cex.lab=1.5,cex.main=1.5, xlab = "", ylab = "")

legend('topleft', c("European badger", "Wild boar"), lty=c(1,2), col=c(1,4), bty='n',cex=1.5)

text(x = 21, y = 0.145, labels = sprintf("Dhat1: 0.60"), col = "black", cex = 1.3)

par(mar = c(3, 3, 2, 1))

###To estimate confidence intervals we need to know the sampling distribution

###which our coefficient of overlapping is drawn from,

###i.e, the distribution we would get if we had a very large number of independent samples from nature.

###The best way to investigate this is to use a bootstrap.

MMboot<-resample(MM,1000) ###2000 times Bootstrap for European badger (MM) by using resample function

SSboot<-resample(SS,1000) ###2000 times Bootstrap for Wild boar (SS) by using resample function

MM.SSboot <- bootEst(MMboot, SSboot) ###Bootstrap estimate for both MMboot and SSboot

BSMM.SS<-colMeans(MM.SSboot) ###Mean of Bootstrap value

BSMM.SS

MM.CAM<-MM.SSboot[,2] #Extract the require column of the matrix

bootCI(rMM.SS[2],MM.CAM)

###Activity Overlap######

### Overlap between Wildcat (FS) and Beech marten (MF)

min(length(FS), length(MF)) #Checking minimum length from both species

rFS.MF <- overlapEst(FS,MF) ###Overlap for both species by using overlapEst function

rFS.MF

#b1 (Dhat1) for lower than 75 observation

#b4 (Dhat4) for higher than 75 observation

#####Drawing overlap plot FS vs MF

overlapPlot(FS,MF, main="",ylim=c(0,0.15),cex.axis=1.5,cex.lab=1.5,cex.main=1.5, xlab = "", ylab = "")

legend('topleft', c("Wildcat", "Beech marten"), lty=c(1,2), col=c(1,4), bty='n',cex=1.5)

text(x = 21, y = 0.145, labels = sprintf("Dhat1: 0.69"), col = "black", cex = 1.3)

par(mar = c(3, 3, 2, 1))

###To estimate confidence intervals we need to know the sampling distribution

###which our coefficient of overlapping is drawn from,

###i.e, the distribution we would get if we had a very large number of independent samples from nature.

###The best way to investigate this is to use a bootstrap.

FSboot<-resample(FS,1000) ###2000 times Bootstrap for Wildcat (FS) by using resample function

MFboot<-resample(MF,1000) ###2000 times Bootstrap for Beech marten (MF) by using resample function

FS.MFboot <- bootEst(FSboot, MFboot) ###Bootstrap estimate for both FSboot and MFboot

BSFS.MF<-colMeans(FS.MFboot) ###Mean of Bootstrap value

BSFS.MF

FS.CAM<-FS.MFboot[,2] #Extract the require column of the matrix

bootCI(rFS.MF[2],FS.CAM)

###Activity Overlap######

### Overlap between Wild boar (SS) and Beech marten (MF)

min(length(SS), length(MF)) #Checking minimum length from both species

rSS.MF <- overlapEst(SS,MF) ###Overlap for both species by using overlapEst function

rSS.MF

#b1 (Dhat1) for lower than 75 observation

#b4 (Dhat4) for higher than 75 observation

#####Drawing overlap plot SS vs MF

overlapPlot(SS,MF, main="",ylim=c(0,0.15),cex.axis=1.5,cex.lab=1.5,cex.main=1.5, xlab = "", ylab = "")

legend('topleft', c("Wild boar", "Beech marten"), lty=c(1,2), col=c(1,4), bty='n',cex=1.5)

text(x = 21, y = 0.145, labels = sprintf("Dhat1: 0.72"), col = "black", cex = 1.3)

par(mar = c(3, 3, 2, 1))

###To estimate confidence intervals we need to know the sampling distribution

###which our coefficient of overlapping is drawn from,

###i.e, the distribution we would get if we had a very large number of independent samples from nature.

###The best way to investigate this is to use a bootstrap.

SSboot<-resample(SS,1000) ###2000 times Bootstrap for Wild boar (SS) by using resample function

MFboot<-resample(MF,1000) ###2000 times Bootstrap for Beech marten (MF) by using resample function

SS.MFboot <- bootEst(SSboot, MFboot) ###Bootstrap estimate for both SSboot and MFboot

BSSS.MF<-colMeans(SS.MFboot) ###Mean of Bootstrap value

BSSS.MF

SS.CAM<-SS.MFboot[,2] #Extract the require column of the matrix

bootCI(rSS.MF[2],SS.CAM)
